# ATP1A2 Mutations in Migraine: Seeing through the Facets of an Ion Pump onto the Neurobiology of Disease

**DOI:** 10.3389/fphys.2016.00239

**Published:** 2016-06-21

**Authors:** Thomas Friedrich, Neslihan N. Tavraz, Cornelia Junghans

**Affiliations:** Department of Physical Chemistry/Bioenergetics, Institute of Chemistry, Technical University of BerlinBerlin, Germany

**Keywords:** familial hemiplegic migraine, Na^+^/K^+^-ATPase, human ATP1A2, neuronal hyperexcitability, protein expression, protein stability, protein targeting, structure-function studies

## Abstract

Mutations in four genes have been identified in familial hemiplegic migraine (FHM), from which *CACNA1A* (FHM type 1) and *SCN1A* (FHM type 3) code for neuronal voltage-gated calcium or sodium channels, respectively, while *ATP1A2* (FHM type 2) encodes the α_2_ isoform of the Na^+^,K^+^-ATPase's catalytic subunit, thus classifying FHM primarily as an ion channel/ion transporter pathology. FHM type 4 is attributed to mutations in the *PRRT2* gene, which encodes a proline-rich transmembrane protein of as yet unknown function. The Na^+^,K^+^-ATPase maintains the physiological gradients for Na^+^ and K^+^ ions and is, therefore, critical for the activity of ion channels and transporters involved neuronal excitability, neurotransmitter uptake or Ca^2+^ signaling. Strikingly diverse functional abnormalities have been identified for disease-linked ATP1A2 mutations which frequently lead to changes in the enzyme's voltage-dependent properties, kinetics, or apparent cation affinities, but some mutations are truly deleterious for enzyme function and thus cause full haploinsufficiency. Here, we summarize structural and functional data about the Na^+^,K^+^-ATPase available to date and an overview is provided about the particular properties of the α_2_ isoform that explain its physiological relevance in electrically excitable tissues. In addition, current concepts about the neurobiology of migraine, the correlations between primary brain dysfunction and mechanisms of headache pain generation are described, together with insights gained recently from modeling approaches in computational neuroscience. Then, a survey is given about ATP1A2 mutations implicated in migraine cases as documented in the literature with focus on mutations that were described to completely destroy enzyme function, or lead to misfolded or mistargeted protein in particular model cell lines. We also discuss whether or not there are correlations between these most severe mutational effects and clinical phenotypes. Finally, perspectives for future research on the implications of Na^+^,K^+^-ATPase mutations in human pathologies are presented.

## Na^+^,K^+^-ATPase in familial hemiplegic migraine and other inherited diseases

Migraine is a particularly disabling pathology with a high cost for human society. The “Atlas of Headache Disorders and Resources in the World 2011” issued by the WHO reported that about 10% of the world's population suffer from migraine, with three times more women affected than men, causing 190 million days lost from work every year in Europe alone, which ranks migraine in the fourth place among neurological disorders with an estimated annual cost of about 116 billion Euros.

Migraine frequently appears with perceptional or somatosensory disturbances, which are called “aura” symptoms. These can include alterations in the field of vision (scotoma), flashes, strange smells or sounds, but also tingling, numbness or partial paresis. Familial Hemiplegic Migraine (FHM) is an autosomal dominantly inherited form of migraine with aura (MA, as opposed to migraine without aura, MO), in which the accompanying aura symptom of the typical half-sided headache is transient motor weakness (hemiparesis) that is frequently accompanied by other cortical symptoms. Four FHM types have been identified by human geneticists, from which FHM type1 (FHM1) and type 3 (FHM3) affect the genes coding for the neuronal voltage-gated P/Q-type calcium channel's α-subunit (*CACNA1A*) (Ophoff et al., 1996) or the neuronal voltage-gated sodium channel's α-subunit (*SCN1A*) (Dichgans et al., 2005), respectively. FHM type 2 (FHM2) is caused by mutations in the *ATP1A2* gene (De Fusco et al., 2003), which encodes the isoform 2 of the human Na^+^,K^+^-ATPase's large catalytic α-subunit, which in the adult central nervous system (CNS) is mainly expressed in astrocytes. Recently, a fourth FHM gene, *PRRT2*, has been identified (Riant et al., 2012), which encodes a proline-rich transmembrane protein of still unknown function. The PPTR2 protein was suggested to interact with the synaptosomal-associated protein 25 (SNAP-25), a t-SNARE protein, which accounts for the specificity and execution of synaptic vesicle fusion with the plasma membrane (Rizo and Südhof, 2002).

Hemiplegic migraine and MA/MO also occur as a comorbidity in proximal renal tubular acidosis (pRTA) patients carrying certain homozygous mutations in the *SLC4A4* gene (encoding the Na^+^-${\text{HCO}}_{3}^{-}$ cotransporter NBCe1), in which mutations in the other known FHM-related genes were ruled out (Suzuki et al., 2010). The NBCe1B splice variant is expressed in several tissues including brain, and its transport activity in astrocytes is thought to modulate neuronal excitability by regulating local pH (Chesler, 2003) suggesting that also defective pH regulation in the brain may be a susceptibility factor in hemiplegic and other types of migraine.

The Na^+^,K^+^-ATPase belongs to the large family of P-type ATPases (Axelsen and Palmgren, 1998). The minimal unit is composed of a large catalytic α-subunit (~1020 amino acids, see Section Functional Insights Gained from Structural Studies) and a smaller, ancillary β-subunit (~300 amino acids, one transmembrane domain (TM) with a heavily glycosylated ectodomain). The β-subunit is a mandatory feature of K^+^-countertransporting P_2C_-type ATPases, which assists in proper folding, assembly and targeting of the holoenzyme (Jaunin et al., 1993), and modulates cation affinities (Crambert et al., 2000). According to molecular modeling studies, the particular β-isoform serves in tuning the pump depending on its individual tilt angle (Hilbers et al., 2016) by differentially stabilizing the E_1_P(3Na^+^) state. There is a still unresolved controversy about the existence of higher oligomeric states (see Donnet et al., 2001; Clarke, 2009; Shattock et al., 2015; and references therein), which, if true, would allow for speculations about possible dominant-negative effects in the heterozygous state of affected patients. Based on earlier biochemical evidence (Forbush et al., 1978), a third, auxiliary γ-subunit was identified (66 amino acids, one TM) (Mercer et al., 1993), which belongs to the class of FXYD-domain containing ion transport regulator proteins (Sweadner and Rael, 2000) and is now classified as FXYD2. The FXYD family, named after the invariant amino acid motif FXYD, comprises seven members in humans (FXYD1, or phospholemman; FXYD2, or Na^+^,K^+^-ATPase γ-subunit; FXYD3, or Mat-8; FXYD4, or corticosteroid hormone-induced factor, CHIF; FXYD5, or “related to ion channel”, RIC, also termed dysadherin; FXYD6, or phosphohippolin; FXYD7), from which all but FXYD6 were shown to associate with Na^+^,K^+^-ATPase α/β-complexes and exerted distinct effects on pump function (see reviews by Garty and Karlish, 2006; Geering, 2006). Since the various FXYD isoforms have different tissue distribution and functional effects, with prominent expression in electrically excitable or fluid- and solute-transporting tissues, these proteins act as tissue-specific modulators of Na^+^,K^+^-ATPase in order to fine-tune its kinetic properties according to the tissue's requirements or physiological state. In the brain, FXYD1, -6, and -7 are the most abundant isoforms (Garty and Karlish, 2006).

Four α-isoforms exist in humans, from which α_1_ is ubiquitously expressed and therefore the most indispensable isoform for cellular ion homeostasis, volume regulation, excitability etc. The α_2_-isoform (ATP1A2) is particularly high expressed in skeletal muscle (SM), but also heart and vascular smooth muscle (VSM) express it. In the adult central nervous system (CNS), α_2_ is mainly found in astrocytes, whereas α_3_ (ATP1A3) is neuronal-specific, and α_4_ has only been found in testis (spermatozoa) (see Blanco et al., 1999; Larsen et al., 2014; Shattock et al., 2015 and references therein). Two other types of human inherited diseases have been linked to α-subunit isoforms. Mutations in the *ATP1A3* gene cause Rapid Dystonia Parkinsonism (RDP, DYT12) (de Carvalho Aguiar et al., 2004), as well as Alternating Hemiplegia of Childhood (AHC) (Heinzen et al., 2012). While the *ATP1A1* gene for the ubiquitously expressed α_1_-isoform is regarded as a susceptibility locus in human essential hypertension (Glorioso et al., 2001), congenital mutations in the gene have not been described so far. However, somatic *ATP1A1* mutations were detected in aldosterone-producing adenomas (APA) and secondary hypertension (Azizan et al., 2013; Beuschlein et al., 2013). Functional studies of the ATP1A1 mutants showed loss of pump activity, strongly reduced K^+^ affinity and augmented inward proton leak currents (see next section) at physiological potentials and concentrations of Na^+^ and K^+^ (Azizan et al., 2013). Furthermore, abnormal depolarization was observed in primary adrenal adenoma cells (Beuschlein et al., 2013), which was suggested to be the consequence of enhanced inflow of protons rather than being caused by reduced Na^+^,K^+^ pumping (Azizan et al., 2013). This may indirectly lead to enhanced Ca^2+^ signaling and, consequently, enhanced aldosterone output (Beuschlein et al., 2013). Another syndrome, renal hypomagnesemia type 2 (HOMG2), an autosomal dominant pathology with isolated renal magnesium loss, is linked to mutations in the γ-subunit's *FXYD2* gene. Evidence has accumulated that *FXYD2* mutations cause misrouting of the Na^+^,K^+^-ATPase, which would lead to loss of plasma membrane protein in the kidney (Meij et al., 2000).

At least indirectly associated with Na^+^,K^+^-ATPase is Long QT Syndrome type 4 (LQT4), a type of cardiac arrythmia with a prolonged QT-phase in electrocardiograms (Lu and Kass, 2010) that frequently causes cardiac fibrillation and sudden death. Unlike other LQT types, LQT4 does not relate to genes of cardiac ion channels, but affects the *ANK2* gene encoding ankyrin-B. Ankyrin-B serves as a scaffold protein responsible for proper targeting of Na^+^,K^+^-ATPase, the Na^+^,Ca^2+^-exchanger and the InsP3 receptor to T-tubules/sarcoplasmic reticulum microdomains in cardiac muscle cells (Mohler et al., 2005). Thus, not only molecular function, but also cellular processing must be considered in the neurobiology of disease.

FHM2 cases frequently share comorbidity with other neurological disorders, such as seizures, AHC and even epilepsy (Supplementary Table 1). This overlap is intriguing, since all these phenomena are linked to deficient regulation of the cortical excitatory/inhibitory balance (Pietrobon and Moskowitz, 2013). Most of the aura symptoms are caused by the phenomenon of Cortical Spreading Depression (CSD) (Leão, 1944) or CSD-like events that are characterized by a spreading front of excitation, which is followed by a long-lasting depression (Moskowitz et al., 2004). Whereas CSD spreads slowly over the neocortex, epilepsy is characterized by rapidly circulating waves of neuronal hyperexcitation. This pathophysiological overlap raises the question, which parameters determine the evolution of such a highly non-linear excitable system as the neocortex into one or the other hyperexcitation pattern (Ullah et al., 2015).

## Na^+^,K^+^-ATPase: function and structure

### Functional properties of the Na^+^,K^+^-ATPase

**Figure 1:**
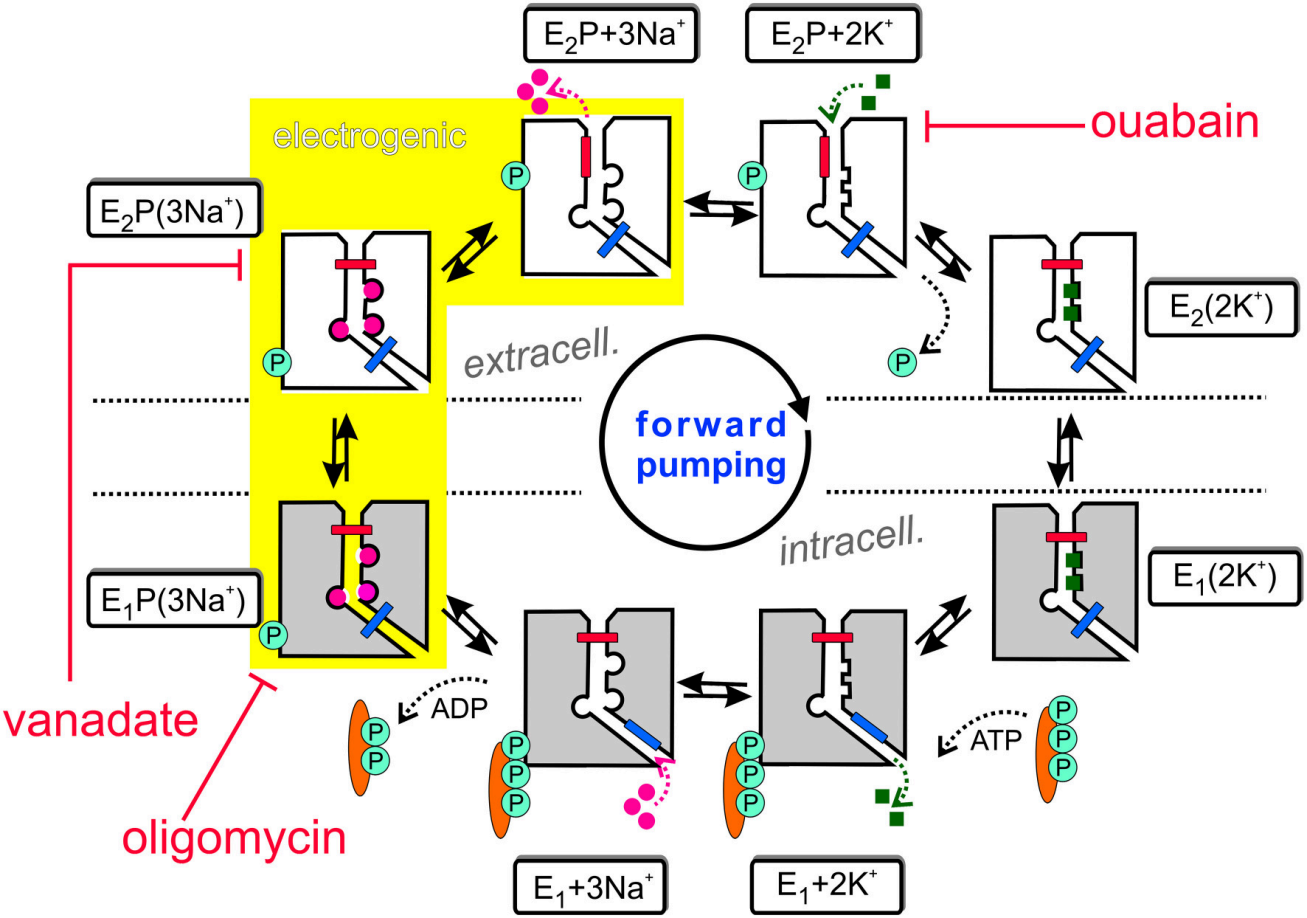
**The Post-Albers reaction mechanism of the Na^**+**^,K^**+**^-ATPase. See text for details**.

The Na^+^,K^+^-ATPase is an electrogenic, primary active transporter protein, which energizes the membrane of all animal cells with the characteristic electrochemical gradients for Na^+^ and K^+^ ions. These gradients are pivotal for the activity of secondary active transporters such as the Na^+^,Ca^2+^-exchanger (NCX), neurotransmitter uptake transporters or voltage-gated Na^+^ and K^+^ channels involved in electrical excitability. The mechanism of function is generally expressed in the form of the Post-Albers scheme (Albers, 1967; Post et al., 1972), as shown in Figure 1. In each reaction cycle, the Na^+^, K^+^-ATPase transports three Na^+^ ions out of and two K^+^ ions into the cell upon hydrolysis of one ATP molecule. The enzyme undergoes cyclic interconversions between two principal conformations, E_1_ and E_2_ and phosphorylated intermediates thereof, E_1_P and E_2_P, in which a phosphate group from ATP is covalently attached to a critical aspartate residue within the S**D**KTGTLT motif (see next section about structural details). Upon binding of three Na^+^ ions from the intracellular side in the ATP-bound E_1_ conformation, the phosphorylated intermediate with three occluded Na^+^ ions, E_1_P(3Na^+^), is formed. This is followed by a conformational change to the E_2_P(3Na^+^) conformation, from which Na^+^ ions are extracellularly released. Because of the increased affinity for K^+^ in this configuration, two K^+^ ions bind subsequently from the extracellular side, which triggers dephosphorylation and occlusion of two K^+^ ions in the E_2_(2K^+^) state. After another conformational change to E_1_(2K^+^), the K^+^ ions dissociate into the cytoplasm, a process that is speeded up by ATP binding.

Also included in Figure 1 are inhibitors of the Na^+^ pump. Ouabain (or g-strophanthin) belongs to the family of cardiotonic steroids, from which digitalis (g-strophantidin) from the red foxglove *Purpurea officinalis* was in medicinal use against dropsy and cardiac insufficiency for centuries (Withering, 1785). Ouabain is used to isolate Na^+^, K^+^-ATPase functional activity in cells or tissue preparations and was a prerequisite for the identification of the “sodium- and potassium-transporting adenoside triphosphatase” by Chemistry Nobel Prize awardee Jens Christian Skou (Skou, 1957). The compound arrests the Na^+^ pump with high affinity (with IC_50_ or K_D_ values in the range of tens of nM for human Na^+^,K^+^-ATPase α_1_-, α_2_-, or α_3_-subunits in complex with human β_1_-subunit, (see Katz et al., 2010; Weigand et al., 2014a) and references therein) by binding from the extracellular side to the E_2_P conformation, and this interaction competes with extracellular K^+^ binding. In contrast, (ortho-)vanadate (${\text{VO}}_{4}^{-}$) blocks the pump from the intracellular side with nanomolar affinity. With its similarity to the phosphate (${\text{PO}}_{4}^{3-}$) anion and its trigonal bipyramidal structure, it serves as a transition state analog for the hydrolytic dephosphorylation of the phosphointermediate. Vanadate arrests the Na^+^ pump in the E_2_P(3Na^+^) form (Glynn, 1985). Oligomycin, a macrolide antibiotic from *Streptomyces*, also inhibits the Na^+^,K^+^-ATPase (Glynn, 1985) by promoting the occlusion of Na^+^ ions in the E_1_P form, and decreases the rate of Na^+^ release from the phosphoenzyme, thereby inhibiting the E_1_P → E_2_P interconversion (Glynn, 1985; Skou, 1990).

The sequential translocation of Na^+^ and K^+^ ions requires strict cation specificity of the phosphorylation and dephosphorylation reactions, mutual changes of the apparent affinities for Na^+^ and K^+^ and alternating access of the cation binding sites to the extra- and intracellular medium. Active transport by an ion pump also requires the operation of two “occlusion gates” (indicated by a blue and red bar in Figure 1) shielding the bound cations from the extra- or intracellular medium, which must never be open simultaneously (Gadsby, 2009). According to the 3Na^+^/2K^+^ stoichiometry, the Na^+^,K^+^-ATPase produces an outward movement of one positive charge per cycle and generates a pump current. The major electrogenic event has been shown to take place during extracellular release (or reverse binding) of Na^+^ (Fendler et al., 1985; Gadsby et al., 1985; Nakao and Gadsby, 1986; Gadsby and Nakao, 1989; Rakowski et al., 1991; Rakowski, 1993; Wuddel and Apell, 1995) within a sequence of coupled partial reactions, which is underlaid in yellow in Figure 1. Electrogenicity arises from passage of Na^+^ ions through a narrow, high-field “access channel” to/from the extracellular space (Läuger, 1979; Gadsby et al., 1993; Hilgemann, 1994; Sagar and Rakowski, 1994; Rakowski et al., 1997; Holmgren et al., 2000; Holmgren and Rakowski, 2006). This term, combined with the notion of its “fractional depth”, is frequently used to denote that an ion passes a certain fraction of the transmembrane electric field in order to reach or exit from its binding site. In the same way as the existence of a positive slope of the stationary current-voltage (I-V) curve in the negative voltage range indicates electrogenic extracellular Na^+^ release (or reverse binding) (Nakao and Gadsby, 1989), the negative slope in the I-V curve at positive voltages (Rakowski et al., 1991) suggests that also K^+^ ions bind within an extracellular access channel, albeit of smaller fractional depth (see Section Electrophysiological Assays: The Two-Electrode Voltage Clamp for more details). There might well be other steps in the catalytic cycle, which contribute to the total electrogenicity of the Na^+^ pump, such as intracellular Na^+^ binding, as shown by Pintschovius et al. (1999) as well as Apell and Karlish (2001).

Electrophysiology has elucidated another functional detail of the Na^+^,K^+^-ATPase, namely the ouabain-sensitive “leak currents”, which were first observed at negative voltages in the absence of extracellular Na^+^ and K^+^, and are augmented by extracellular acidification. Initially reported by Rakowski et al. (1991) and later investigated in more detail by Efthymiadis et al. (1993), Wang and Horisberger (1995), Rettinger (1996) and Li et al. (2006), this property has for a long time been merely recognized as a footnote in Na^+^ pump research, until structural and functional evidence highlighted the critical role of protons in the transport cycle, as indicated by mutations that interfere with a C-terminal pathway for protons (see Section Functional Insights Gained from Structural Studies) to access the cation binding pocket (Morth et al., 2007; Poulsen et al., 2010). Recently, it was demonstrated that the proton leak inward current is a property inherent to Na^+^,K^+^-ATPase that also flows at physiological K^+^ and Na^+^ concentrations and membrane potentials (Mitchell et al., 2014; Vedovato and Gadsby, 2014), even in native cells, i.e., in the presence of the pump's normal regulatory subunit phospholemman, which is prevalent in cardiac tissue (Mitchell et al., 2014). Vedovato and Gadsby concluded that inward proton leak exploits the reversibility of a subset of conformational changes associated with extracellular Na^+^ release from the phosphorylated enzyme. Although such a back-step of phosphorylated Na^+^,K^+^-ATPase that enables proton import is not required for completion of the 3Na^+^/2K^+^ transport cycle, it readily occurs during Na^+^, K^+^ transport when external K^+^ ion binding and occlusion are retarded, and it occurs more frequently when the probability for extracellular proton access is increased by acidification (Vedovato and Gadsby, 2014). The protons presumably pass through the Na^+^-selective binding site III (which may be in fact two sites, see Section Functional Insights Gained from Structural Studies) via the carboxylates of Glu-958 to Asp-930 *via* the intervening hydroxyl of Tyr-775 (ATP1A2 numbering), which is distinct from the principal pathway of the Na^+^ and K^+^ ions passing through binding site II. From the simultaneous occurrence of Na^+^, K^+^ exchange and H^+^ import during the same conformational cycle of a single molecule, the Na^+^,K^+^-ATPase classifies as a hybrid transporter, although the physiological or pathophysiological significance of pump–mediated proton inflow still has to be clarified (Vedovato and Gadsby, 2014). By performing experiments over an expanded range of extracellular pH (pH_o_) values and meticulous ion competition assays, Mitchell et al. delineated a previously unrecognized inhibitory action of extracellular protons on the leak current, which occurs in addition to the pH_o_-induced leak stimulation (Mitchell et al., 2014). Based on the strong voltage dependence of the pH_o_-induced leak stimulation, these authors concluded that protons leak through the site responsible for strong Na_o_- and voltage-dependent inhibition of pump current, which must be site III, in agreement with (Vedovato and Gadsby, 2014). The pH_o_-dependent inhibition of the leak, although profoundly enhanced by decreasing pH_o_, was nevertheless only weakly voltage-dependent, similar to the inhibition by ${\text{Na}}_{\text{o}}^{+}$ or extracellular K^+^ binding, suggesting that inhibition of the leak current by external cations (H^+^, Na^+^ or K^+^) requires binding to sites I and II that are not responsible for the Na^+^,K^+^-ATPase's voltage dependence (Mitchell et al., 2014).

The inward current was found to be augmented by mutations at the Na^+^,K^+^-ATPase's C-terminus (Yaragatupalli et al., 2009; Meier et al., 2010; Poulsen et al., 2010; Vedovato and Gadsby, 2010; Paulsen et al., 2012), and sometimes further enhanced with increasing extracellular [Na^+^], which conforms with an earlier proposal that Na^+^ ions might flow along what has been referred to as the Na^+^,K^+^-ATPase “leak” pathway, a process that is promoted by extracellular protons (Vasilyev et al., 2004). The studied C-terminal mutations frequently correlate with drastic decreases in the apparent affinity for extracellular and intracellular Na^+^ (Toustrup-Jensen et al., 2009, 2014), because C-terminal mutations interfere with Na^+^ binding site III.

Whether or not Na^+^,K^+^ pump-mediated proton uptake plays a physiological or pathological role is still unclear. Vedovato and Gadsby pointed out, that already at physiological pH_o_, and even more so at decreased pH_o_ in a pathophysiological situation, the Na^+^, K^+^ pump will import one or several protons downhill in each ATPase transport cycle. Thus, proton inward leak might significantly accompany Na^+^, K^+^ pumping at the normal negative resting potentials of neurons as well as cardiac and skeletal muscle cells, if extracytoplasmic pH became sufficiently low, as e.g., during vigorous muscle exercise, or in cardiac or cerebral ischemia, since Na^+^,K^+^-ATPase densities in muscle, nerve, and heart can be very high (≥1000 μm^2^). It is also conceivable that the known limitation of H^+^-ATPase-mediated acidification of early endosomes by endocytosed Na^+^,K^+^-ATPase might be the direct consequence of Na^+^,K^+^ pump-mediated flow of protons from the endosome lumen to the cytoplasm (Vedovato and Gadsby, 2014). The importance of cortical pH regulation is underlined by the involvement of certain homozygous mutations in the NBCe1 Na^+^-${\text{HCO}}_{3}^{-}$ cotransporter, which is expressed in astrocytes, in pRTA patients that additionally suffer from hemiplegic migraine (Suzuki et al., 2010). Glial cell depolarization usually results from elevated extracellular [K^+^], which causes glial cell acid secretion via inward electrogenic Na^+^-${\text{HCO}}_{3}^{-}$ cotransport, resulting in depolarization-induced alkalosis (DIA) in the cytoplasm of glial cells (Chesler, 2003). The extracellular acidosis that occurs simultaneously to DIA makes the surrounding neuronal cells less excitable because excitatory NMDA receptors are blocked by protons (Suzuki et al., 2010). Therefore, extracellular acidosis suppresses neuronal excitability, and the reduction of DIA due to enhanced proton inward transport by mutant glial Na^+^,K^+^-ATPase could also create a positive feedback loop of increased neuronal activity leading to further NMDA-mediated neuronal hyperactivity, depolarization of brain cells, and CSD (Suzuki et al., 2010). Although increased proton leak has only been reported for the R937P mutation in ATP1A2 (Poulsen et al., 2010), this may not be an isolated observation, since no other ATP1A2 allele has so far been scrutinized for altered proton leak.

### Functional insights gained from structural studies

For the Na^+^,K^+^-ATPase, high-resolution structures of different reaction cycle intermediates are available, which provided an atomic-level understanding of active cation transport coupled to enzymatic catalysis. Structures include the Rb^+^-occluded E_2_P-like conformation $[{\text{Rb}}_{2}^{+}]{\text{E}}_{2}\cdot {\text{MgF}}_{4}^{2-}$ (Morth et al., 2007), which was further refined in the E_2_·2K^+^·P_i_ (also stabilized by ${\text{MgF}}_{4}^{2-}$) structure that revealed the full arrangement of the β-subunit (Shinoda et al., 2009), the low-affinity ouabain- and 2K^+^-bound E_2_·2K^+^·P_i_ (also stabilized by ${\text{MgF}}_{4}^{2-}$) structure (Ogawa et al., 2009), the high-affinity ouabain-bound E_2_P-like state with Mg^2+^ bound to the cation binding pocket (Ogawa et al., 2009), the Na^+^-bound E_1_·(${\text{AlF}}_{4}^{-}$)·ADP·3Na^+^ structure (stabilized with ${\text{AlF}}_{4}^{-}$) of an intermediate preceding the Na^+^-occluded E_1_P(3Na^+^) state (Kanai et al., 2013), and a comparable [${\text{Na}}_{3}^{+}$]E_1_P-ADP state in complex with ${\text{AlF}}_{4}^{-}$, with Na^+^ saturation stabilized by oligomycin (Nyblom et al., 2013). These structural data, together with the wealth of intermediate structures determined for the related SERCA Ca^2+^-ATPase from sarcoendoplasmic reticulum (Olesen et al., 2007), provide a comprehensive concept of the catalytic mechanism carried out by the Na^+^ pump.

**Figure 2:**
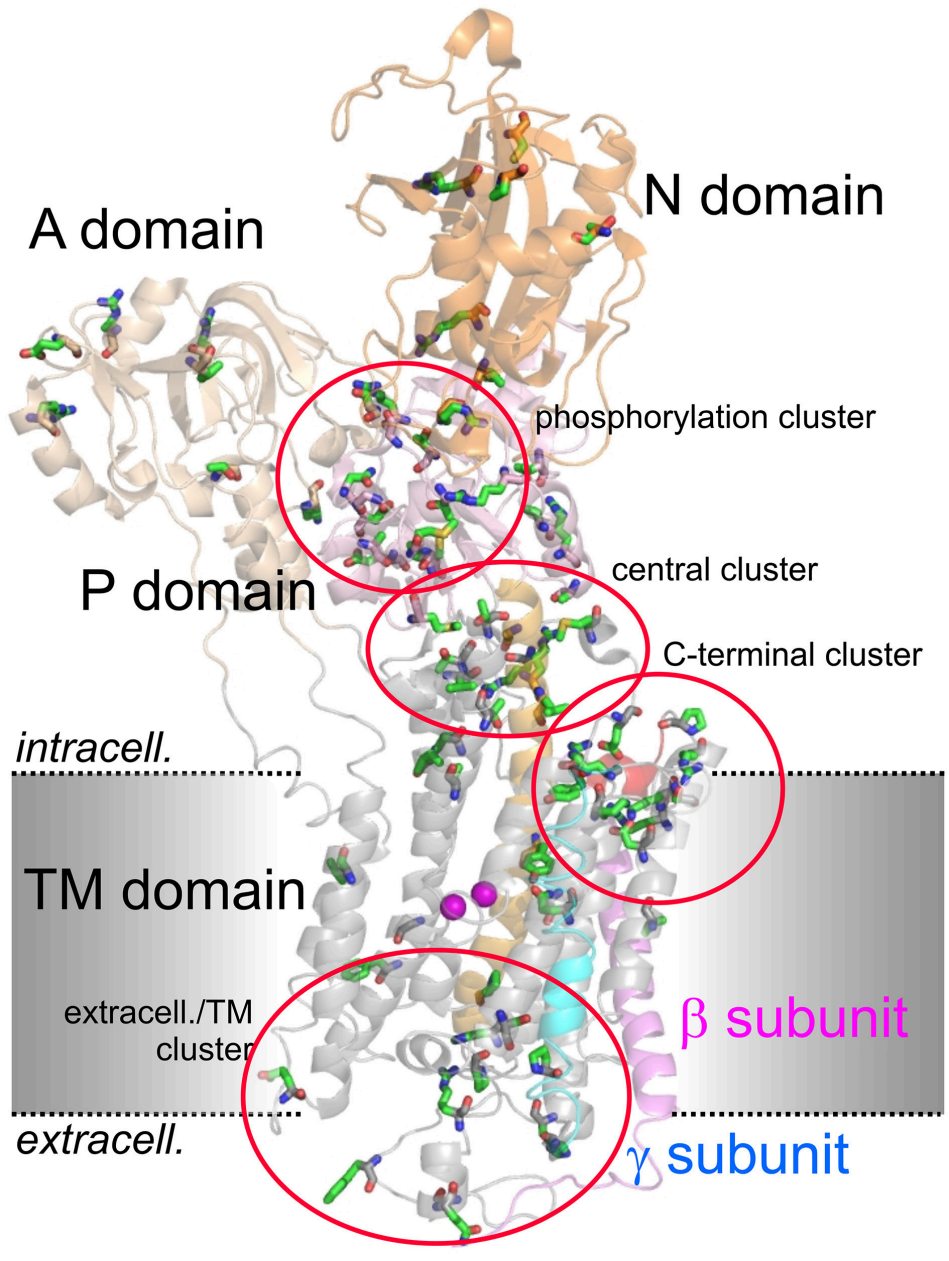
**Structure of the Na^**+**^,K^**+**^-ATPase (PDB code 3B8E; Morth et al., 2007) and location of migraine-associated ATP1A2 mutations**. The domains of the cytoplasmic part are A (actuator; light orange), N (nucleotide binding; orange) and P (phosphorylation; pink) domain, transmembrane helices are depicted in gray, except for the about 70 Å-long central TM5 helix (orange). The β- and γ-subunits are shown in magenta and blue, respectively, two Rb^+^ ions in the cation binding pocket are shown as purple spheres. Amino acids mutated in migraine cases as listed in Supplementary Table 1 are shown in stick representation. More than 80% of mutations fall into four clusters, one around the catalytic P domain, one in a central region between P and TM domain, one within the extracellularly-facing part of the TM domain, and one around the enzyme's C-terminus.

Figure 2 shows the arrangement of the α-, β- and γ-subunit of the Na^+^,K^+^-ATPase in the 2Rb^+^-bound E_2_P-like conformation (Morth et al., 2007). Several distinct domains can be distinguished on the α-subunit, with 10 TM segments forming the TM domain harboring the coordination sites for cations and the central TM5 helix, which extends into the central P (phosphorylation) domain. The P domain harbors the P-type ATPase consensus motif ^373^S**D**KTGTLT^380^ with the intermediately phosphorylated Asp-374 (all numbering refers to human ATP1A2), which is surrounded by the ^612^MVTGD^616^ and ^713^DG(V/M)ND^717^ motifs and the critical Lys-605. Nucleotide binding occurs in the N domain, and the A (actuator) domain serves as an anchor for the movement of the N domain by performing a hinge-like movement during the conformational cycle to bring the conserved ^217^TGES^220^ motif into close proximity with ^612^MVTGD^616^ to expedite dephosphorylation. Movement of the A domain also entails a piston-like movement of TM helices 1 and 2. The crystal structures also highlighted a particularly crucial arrangement of the enzyme's C-terminal sequence ^1013^WVEKETYY^1020^. The first part of this motif assumes an α-helical structure accommodated between the TM of the β-subunit and the TM7 and TM10 helices, and the two terminal tyrosines project into a binding pocket between TM7, TM8, and TM5, with the terminal Tyr-1020 interacting with Lys-770 on TM5 and Arg-937 in the loop connecting TM8 and TM9 (Morth et al., 2007). Deletion of the terminal KETYY sequence resulted in a 26-fold reduction of Na^+^ affinity, reminiscent of the effect of mutations of putative Na^+^ coordinating residues. This has prompted investigations of the functional importance of C-terminal residues by studying deletions, mutations of the terminal tyrosines, or C-terminal extensions. These studies showed that alterations in the C-terminal sequence entail drastic decreases in Na^+^ affinity and enhance the propensity of the mutant pumps to permit inward proton leak (Yaragatupalli et al., 2009; Meier et al., 2010; Poulsen et al., 2010; Vedovato and Gadsby, 2010, 2014; Paulsen et al., 2012). This has given rise to the concept that the C-terminal pathway occupied by the terminal tyrosines defines a mandatory access route for intracellular protons to the Na^+^ binding site III to bring about stoichiometric 3Na^+^/2K^+^ transport, in which Arg-937 and Asp-923 play a critical role (Poulsen et al., 2010).

From the two “common” cation binding sites, site I is made up from five oxygen atoms (from Thr-776 main chain, and from Ser-779, Asn-780 (all TM5), and Asp-808 (TM6) side chains), and one water molecule, whereas site II is coordinated by the main chain carbonyls of Val-327, Ala-328, and Val-330 (all TM4) and the side chain oxygens of Asn-780, Glu-783 (TM5) and Asp-808 (TM6), and possibly Glu-332 (TM4) (Shinoda et al., 2009). Of note, the TM5 helix is unwound at Asn-780 to create sufficient space for cation coordination. The unwinding is due to Pro-782; both amino acids reside in the highly conserved ^779^S**N**I**P**E^783^ motif. In the two E_1_·P·ADP·3Na^+^-like structures, the geometry for Na^+^ coordination at the two “common” sites is essentially the same, with similar TM5 helix unwinding. Regarding the location of Na^+^ binding site III, the structure by Kanai et al. (2013) identified two slightly different locations in the two protomers of the crystallographic unit, and the study by Nyblom et al. (2013) had to resolve a similar ambiguity by proposing two sites, IIIa and IIIb, from which site IIIa is surrounded by Glu-958, Tyr-775, and Thr-811, and site IIIb adjacent to Thr-778, Gln-858, Gln-927, and Asp-930, with a sufficiently clear electron density to identify IIIb as primary Na^+^ binding site III. Mutational analysis and electrophysiology suggested that site IIIa is a transient Na^+^ binding site during extracellular Na^+^ release. The latter was also inferred from proton leak currents of mutant enzymes (see below), which showed that site IIIa mutations (Y775F, Q958A) permitted leak currents in the absence of extracellular Na^+^ but with Na^+^ closing the leak, whereas site IIIb mutations (D930E and Q858N) permitted leak currents with and without Na^+^. This gave rise to the concept that protonation of Asp-930 is associated with voltage-dependent release of Na^+^ from site IIIb via site IIIa to the extracellular space. The proton leak current can occur under conditions when either no Na^+^ ions are bound or when only the two common sites are occupied by Na^+^ ions (Nyblom et al., 2013), or, as later shown, by protons (Mitchell et al., 2014). Then, site IIIa is accessible to an extracellular proton, which upon application of negative voltage can move via site IIIb to the cytoplasm, thus making site IIIa accessible for an extracellular proton again, which eventually leads to a sustained inward proton leak current. This notion was corroborated and refined by two comprehensive electrophysiological studies (Mitchell et al., 2014; Vedovato and Gadsby, 2014).

## The headache of ATP1A2 mutations: neurobiology of migraine

**Figure 3:**
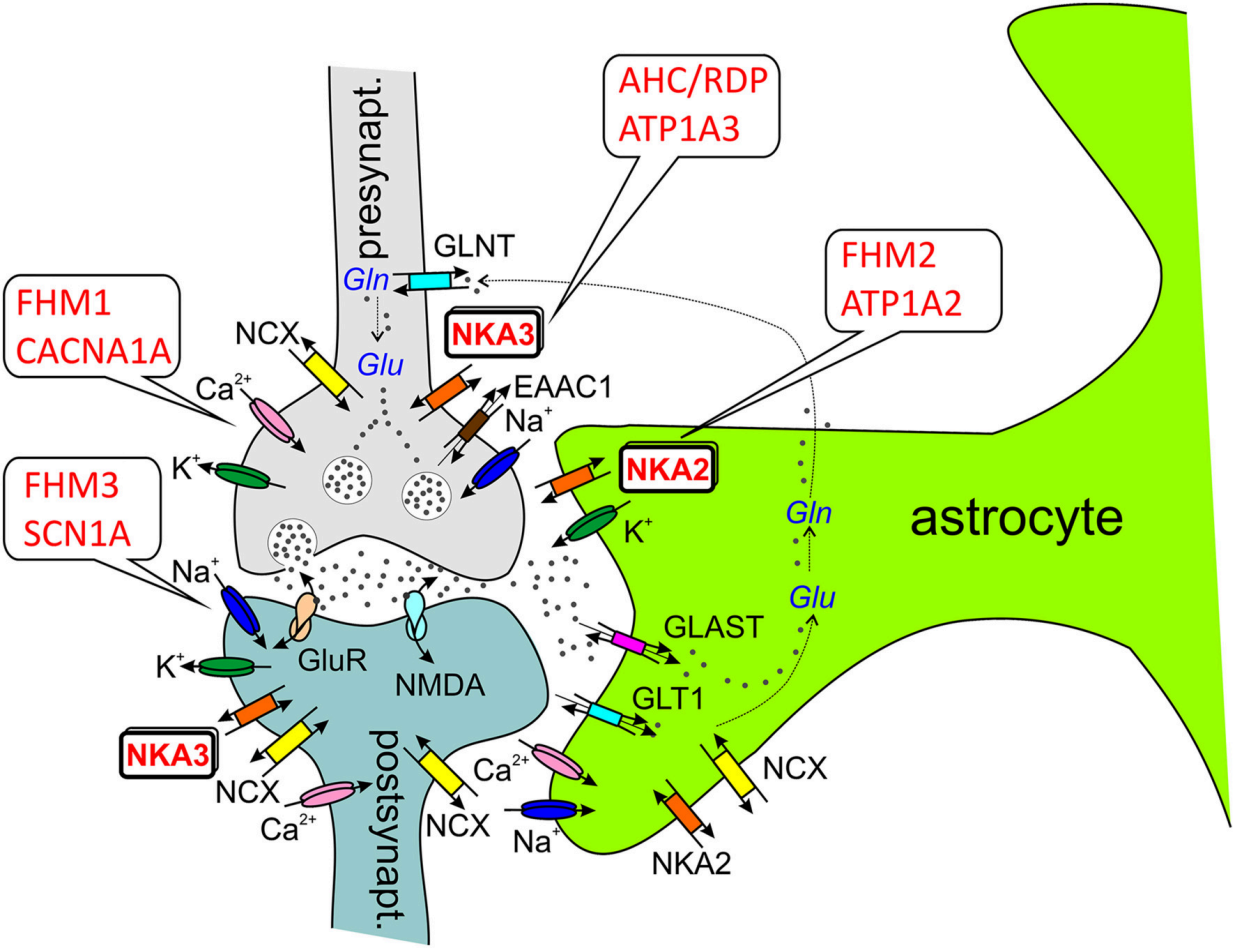
**ATP1A2 and ATP1A3 in the tripartite synapse (Perea et al., 2009)**. Most glutamatergic synapses are in contact with astrocytic processes, which express a high density of excitatory amino acid transporters (EAATs, e.g., GLT1, GLAST), which are crucial for the synaptic glutamate (Glu) clearance. In astrocytes, glutamate is converted to glutamine by glutamine synthetase as part of glutamate recycling. Ion channels involved in electrical excitation are indicated by the respective cations, Na^+^, K^+^, and Ca^2+^. Abbreviations: GLAST (EAAT1), glutamate/aspartate transporter; GLT1 (EAAT2), glutamate transporter; GLNT, glutamine transporter; GluR, (metabotropic) glutamate receptor; NKA2/3, Na^+^, K^+^-ATPase α_2_-α_3_-subunit; NMDA, ionotropic glutamate receptor; NCX, Na^+^,Ca^2+^-exchanger; Gln, glutamine.

How can Na^+^,K^+^-ATPase dysfunction be linked to migraine? In the case of *CACNA1A* and *SCN1A* mutations, functional disturbances are typically gain-of-function effects, and mutated channels, e.g., exhibit an abnormal residual activity in the inactivated state, which leads to prolonged cation influx into neurons, thus causing, *inter alia*, depolarization and lowering of the activation threshold. Whether ATP1A2 mutations in FHM2 classify as gain- or loss-of-function phenomena is not that clear-cut. While many mutations abolish or largely reduce Na^+^,K^+^ pumping implying loss-of-function, others are characterized by subtle changes in voltage dependence (Supplementary Table 1). In these cases, a shift of the pump current's I-V curve may entail loss-of-function in a particular voltage range, but gain-of-function in another. From the viewpoint of Na^+^,K^+^-ATPase cation transport, a first clue is obtained from the fact that hyperkalemia is a known CSD and migraine trigger. Since the transporter in neurons or astrocytes removes extracellular K^+^, dysfunction of the pump can lead to elevated extracellular K^+^ levels, which cause depolarization and reduce the activation threshold. But also Na^+^ extrusion by the enzyme is critical, since, e.g., the activity of secondary active transporters depends on the Na^+^ gradient across the membrane. First, the Na^+^,Ca^2+^-exchanger (NCX1) utilizes Na^+^ import for the extrusion of Ca^2+^, and failure to efficiently remove cytosolic Ca^2+^ during neuronal activity leads to Ca^2+^ accumulation in intracellular stores, from which it is then more heavily released during an action potential, and again, hyperexcitability is the outcome. Second, excitatory neurotransmitter uptake transporters (EAATs) also couple to the Na^+^ gradient, and failure to remove neurotransmitters such as glutamate from the synaptic cleft again results in sustained hyperexcitability (see Figure 3).

It is generally accepted that cortical spreading depression (CSD) is the neurophysiological correlate of migraine aura (Pietrobon and Striessnig, 2003). Therefore, neurobiologists split up the question concerning the pathogenic mechanism of migraine into two parts: (1) What is the relation of ATP1A2 mutations to CSD, and (2), what are the links between CSD and cortical pain perception? Although the existence of a “migraine generator” in the brainstem has not been completely ruled out (Pietrobon and Moskowitz, 2013), there is currently no doubt, that CSD plays a central role in the pathophysiology of migraine.

### From ATP1A2 mutations to CSD: the glutamatergic hypothesis

From clinical studies, it is known that neurophysiological abnormalities in sensory information processing (which are most intense 12–24 h before a migraine attack during the premonitory phase, but disappear a few hours before or during the attack) change in intensity in temporal relation to the migraine episode. This suggests that some intrinsic mechanism in the brains of migraineurs progressively increases the dysfunction in central information processing and the susceptibility to a migraine trigger. These mechanisms may lead to the premonitory symptoms and, above a certain threshold of cortical dysfunction in response to migraine triggers, may eventually ignite CSD. Depending on the study, the cortex of migraineurs is hyperexcitable as a consequence of either enhanced excitation or reduced inhibition, or is hypoexcitable, or has a lower preactivation level. Thus, also here, rather than merely hypo- or hyperexcitability, defective regulation of cortical excitability and the consequently reduced ability to maintain the cortical excitatory/inhibitory (E/I) balance appears to underlie abnormal sensory processing (Pietrobon and Moskowitz, 2013, 2014).

The analysis of experimental CSD in FHM knockin mouse models suggests that CSD is a key migraine trigger, since both FHM1 and FHM2 knockin mice showed a lower electrical stimulation threshold for CSD induction and faster CSD propagation (van den Maagdenberg et al., 2004, 2010; Leo et al., 2011). In FHM1 knockin mice carrying the mild R192Q mutation or the severe S218L mutation in CACNA1A, the strength of CSD facilitation as well as the severity of the subsequent neurological motor deficits and the propensity of CSD to propagate into subcortical structures correlated with the strength of the gain-of-function of the CACNA1A channel and the severity of the clinical phenotype (van den Maagdenberg et al., 2004, 2010). Interestingly, the velocity of propagation and the frequency of CSDs elicited by local application of high [K^+^] were larger in female than in male FHM1 mouse mutants, in correspondence with the higher migraine prevalence of females (Eikermann-Haerter et al., 2009). However, such gender differences were not found in FHM2 knockin mice carrying the (heterozygous) W887R mutation in ATP1A2 (Leo et al., 2011). More importantly, the analysis of cortical synaptic transmission in FHM1 knockin mice revealed differential effects of FHM1 mutations at excitatory and inhibitory synapses: Excitatory synaptic transmission on cortical pyramidal cells was enhanced as a consequence of increased action potential-evoked Ca^2+^ influx and increased glutamate release, and enhanced short-term synaptic depression during trains of action potentials was observed. In contrast, inhibitory neurotransmission at cortical fast spiking interneuron synapses was not altered in FHM1 knockin mice, although being initiated by P/Q-type Ca^2+^ channels as well (Tottene et al., 2009). Although these considerations were restricted to specific cortical subcircuits, the differential effect of FHM1 mutations on excitatory and inhibitory neurotransmission may produce hyperexcitation in certain brain conditions, but may leave the excitatory/inhibitory balance intact in others, consistent with the episodic nature of the disease (Pietrobon and Moskowitz, 2013). The gain-of-function of glutamate release at synapses of cortical pyramidal cells can explain the facilitation of experimental CSD in FHM1 knockin mice, which supports a model of CSD initiation, in which CACNA1A-dependent release of glutamate from cortical pyramidal cell synapses and activation of NMDA receptors play a key role in the positive feedback cycle that ignites CSD (Pietrobon and Moskowitz, 2013). It was suggested that excessive NMDA receptor-mediated glutamatergic transmission following impaired clearance of glutamate by astrocytic processes surrounding glutamatergic synapses (the “tripartite synapse” see Figure 3), where the α_2_ Na^+^,K^+^-ATPase is functionally coupled to glutamate transporters (Cholet et al., 2002; Rose et al., 2009), may underlie the enhanced CSD susceptibility in the FHM2 mouse model (Leo et al., 2011). This has given rise to the “glutamatergic” hypothesis. In the nervous system, the Na^+^,K^+^-ATPase α_2_-isoform is expressed primarily in neurons during embryonic development and at birth, but almost exclusively in astrocytes in the adult (Moseley et al., 2003). Whereas neurons express α_1_- and α_3_-subunits (with distinctly different subcellular localization pattern, Juhaszova and Blaustein, 1997), astrocytes express α_1_- and α_2_-subunits, and studies on primary cultured rat astrocytes suggest, that the contribution of α_2_ to extracellular K^+^ clearance is about 30% of total Na^+^,K^+^-ATPase activity (Larsen et al., 2014). However, α_1_ and α_2_ are differentially distributed in astrocytes. Whereas α_1_ is evenly present at the plasma membrane, α_2_ rather shows a reticular distribution, like the Na^+^,Ca^2+^-exchanger NCX1, where it may play an important role in Ca^2+^ signaling (Juhaszova and Blaustein, 1997; see Section ATP1A2 and Ca^2+^ Signaling), and it was found to be heavily present in glial leaflets surrounding dendritic spines and axo-dendritic synapses, where it colocalizes with GLAST and GLT-1 glutamate transporters (Cholet et al., 2002). This functional link to glutamate transporters in astrocytic processes surrounding glutamatergic synapses suggests specific roles in the regulation of glutamate clearance (Pietrobon, 2007; Pietrobon and Moskowitz, 2013). However, ATP1A2 could have a crucial role in extracellular K^+^ clearance by astrocytes (Larsen et al., 2014), similar to VSM (DiFranco et al., 2015) and cardiomyocytes (Stanley et al., 2015). Due to the stronger voltage-dependent inhibition of α_2_ pumps (which is even augmented for the glial α_2_/β_2_ subunit composition compared to α_1_/β_1_ pumps) and the lower extracellular K^+^ affinity, α_2_ pumps are essentially inactive at the normal, negative resting potential and at normal extracellular [K^+^], whereas it will be fully activated during cell depolarization and elevated K^+^. This provides a substantial reserve pumping activity for K^+^ clearance during strong cortical activity (Larsen et al., 2014). Yet, although ATP1A2 is a key player in K^+^ clearance, this aspect has been considered less important for the pathophysiology of migraine because the duration of the CSD was not prolonged in the FHM2 mouse model (Leo et al., 2011). These findings collectively suggest that ATP1A2 mutations in migraine primarily cause a disorder of glutamatergic neurotransmission with defective regulation of the E/I balance in the brain (Pietrobon and Moskowitz, 2013).

### From CSD to trigeminovascular nociception: the neuroinflammatory hypothesis

CSD can be triggered by local elevations of extracellular [K^+^] as a consequence of the hyperactivity of neuronal circuits in the cerebral cortex. Of note, CSD is a slowly propagating wave of strong neuronal and glial depolarization accompanied by depression of electroencephalographic (EEG) activity and by a large increase in extracellular [K^+^] (Pietrobon and Striessnig, 2003). To explain pain generation, the so-called vascular theory prevailed in the past, which proposed that abnormal dilation of meningeal and/or extracranial arteries causes pain, since these are the only cerebral structures endowed with primary pain receptors. However, clinical and experimental evidence rendered this hypothesis implausible, since vasodilation is neither necessary nor sufficient to cause migraine pain (Pietrobon and Striessnig, 2003). It currently emerges that the migraine headache depends on the activation and sensitization of trigeminal nociceptors that innervate the meninges and their large blood vessels. Mediators of noxious pain, such as protons, nitric oxide, arachidonic acid, and serotonin, besides glutamate and other neurotransmitters, are released during CSD. The neuroinflammatory hypothesis suggests that these substances may activate trigeminal nociceptors innervating blood vessels in the *pia mater*, and, via axon collaterals, dural trigeminal afferents and/or may slowly access the meningeal afferents after disruption of the blood-brain barrier, thus eventually activating central trigeminovascular neurons in the trigeminocervical complex. Activation of the meningeal afferents leads to release of proinflammatory vasoactive neuropeptides, e.g., the calcitonin gene-related peptide (CGRP). These processes may promote “sterile” neurogenic inflammation in the *dura mater* and sustain the activation or sensitization of the trigeminovascular afferents (Pietrobon and Striessnig, 2003).

### ATP1A2 and Ca^2+^ signaling

The concerted action between Na^+^,K^+^-ATPase and NCX1 is particularly important, since α_2_- and α_3_-isoforms (but not α_1_) were found to co-immunoprecipitate with NCX1 in rat brain membrane preparations. This, together with co-localization studies by immunocytochemistry suggested that plasma membrane microdomains containing NCX1 and Na^+^ pumps with α_2_- or α_3_- subunits in neurons and astrocytes form Ca^2+^ signaling complexes with plasma membrane-subjacent “junctional” endoplasmic reticulum (jER) microdomains containing ryanodine receptors and sarco/endoplasmic reticulum Ca^2+^-ATPase (Lencesova et al., 2004), as found previously for the junctional sarcoplasmic reticulum (jSR) microdomains in VSM cells as well as astrocytes (Juhaszova and Blaustein, 1997). In line with this notion, it was shown on primary cultured astrocytes from wild-type α_2_(+/+), knockout α_2_(−/−), and α_2_(+/−) heterozygous mouse fetuses that graded loss of ATP1A2 activity successively increases Ca^2+^ signaling (Golovina et al., 2003). The typical volume of such a jS/ER or “PLasmERosome” (Blaustein and Golovina, 2001) compartment is sub-femtoliter in size, in which 1000 ions already account for micromolar concentrations. As a consequence, a 50% loss in Na^+^ pump activity may indeed be a matter of concern already on short time scales.

### Beyond the glutamatergic hypothesis: cell volume as a control parameter?

One aspect attracting the attention of neurophysiologists is the importance of cell volume changes that inevitably accompany the massive changes in ion concentrations during seizures, spreading depression, or anoxic depolarization. During CSD, extracellular [K^+^] increases to 30–60 mM, but extracellular [Na^+^] and [Cl^−^] decrease to 50–70 mM (Pietrobon and Moskowitz, 2014). The concomitant massive uptake of Na^+^ and Cl^−^ by the Na^+^,K^+^,2Cl^−^ transporter (NKCC1) of astrocytes and simultaneous water uptake through aquaporins leads to astrocyte swelling (Larsen et al., 2014). In this respect, it is interesting to note that CSD can be optically monitored in cortical preparations by measuring the so-called intrinsic optical signal (IOS), a neuroimaging technique that measures cortical reflectance changes with high temporal and spatial resolution. The parameters, which the IOS is sensitive to, are changes in light scattering, blood volume, oxy-/deoxyhemoglobin balance and cytochrome oxidation (Ba et al., 2002). Light scattering (monitored at 850 nm) is particularly sensitive to cell volume changes, and this signal component coincides with the electrical signal of the spreading wavefront. New modeling tools and concepts in computational neuroscience have recently identified cell volume as a critical control parameter that separates CSD from seizures as well as other types of spreading depolarization (Wei et al., 2014; Ullah et al., 2015). These approaches use stunningly sophisticated models of neuronal excitability based on a Hodgkin-Huxley-type framework of differential equations that includes the activity of Na^+^,K^+^ ion pumps (Cressman et al., 2009; Ullah et al., 2009), conservation of particles and charge, and accounts for the energy required to restore ionic gradients (Dahlem et al., 2013; Ullah et al., 2015). According to Larsen et al. (2014), K^+^-induced swelling of astrocytes is mediated by NKCC1, but NKCC1 does not contribute to extracellular K^+^ clearance, an activity exclusively spared for α_2_-containing Na^+^,K^+^-ATPase. Moreover, these authors found indications that glial Na^+^,K^+^-ATPase acts to dampen cell swelling during clearance of stimulus induced [K^+^]. So the action of glial Na^+^,K^+^-ATPase could be four-fold: (1) direct astrocytic K^+^ buffering and clearance, (2) glutamate clearance (indirect *via* glutamate transporters), (3) osmolyte transport to dampen cell swelling, and (4) direct repolarizing activity due to electrogenic charge transport. Given the emphasis that computational neuroscientists currently place on cell volume as a control parameter discriminating between seizures and CSD (Ullah et al., 2015), it will be rewarding to study the relation between Na^+^ pump dysfunction and cell volume regulation in the CNS more closely by experiment and theory.

Computational neuroscientists outlined that the premonitory symptoms of a migraine attack, such as the abnormal responses to food, stress, or light, instead of mistaking them as trigger factors themselves, should rather be considered as indicators of a systemic transition at a culminating point that follows some universal pattern, which is determined by dynamic network biomarkers (DNBs) (Dahlem et al., 2013, 2014). In contrast to traditional biomarkers (e.g., biochemical substances) that are statically enhanced or increased in the pathological state, DNBs are dynamical features of biological networks (also substances or, in general, signals), which, though highly fluctuating, are strongly correlated only during the premonitory phase. Such DNBs characterizing the premonitory period are known for lung injury disease, liver and lymphoma cancer, but still need to be identified for migraine. However, their identification could help to develop strategies for early therapeutic intervention. A new scientific discipline, translational computational neuroscience, which still needs to be inaugurated by close interactions between clinicians, experimentalists and theoreticians, may fuse dynamical systems theory with control theory in order to drive innovations in therapeutic brain stimulation to treat neurological diseases based on theoretical concepts (Dahlem et al., 2013).

## The weal and woe of having Na^+^,K^+^-ATPase α_2_-subunits in certain tissues

What is the advantage of having different Na^+^,K^+^-ATPase α-isoforms expressed in a tissue-specific manner, and which other pathophysiological effects could be expected in the case of α_2_-subunit haploinsufficiency besides the migraine phenotype? Whereas the α_1_- isoform is ubiquitously expressed and most indispensable for the organism, the α_2_- isoform is expressed mainly in heart, skeletal, and vascular smooth muscle, brain, lung, and adipocytes. The α_3_- isoform occurs mainly in neurons and ovaries, as well as in developing hearts of rat and in adult human heart and in white blood cells, and α_4_ is found in sperm, where it is required for sperm motility (see Lingrel, 2010, and references therein). The possibility to coassemble with three β-isoforms and up to seven FXYD proteins adds up further complexity. The tissue and subcellular distribution of the α_2_-isoform, in particular its selective expression in electrically excitable cells, or the cells that surround them, or in VSM suggests that α_2_ could also modulate excitability and contractility in heart and skeletal muscle as well as in the vasculature (Radzyukevich et al., 2004). The functional properties that distinguish α_2_- from α_1_-containing Na^+^ pumps define the importance of this isoform in muscle (DiFranco et al., 2015; Stanley et al., 2015) and astrocytes (see Section From ATP1A2 Mutations to CSD: The Glutamatergic Hypothesis). Stanley et al. showed recently that the unusually steep voltage dependence of ion transport of α_2_-containing Na^+^ pumps in the range of physiological potentials, which is even exacerbated by assembly with β_2_, provides a strong reservoir of pumping activity during the cardiac action potential, while keeping it inactive at normal resting potentials. In addition, as earlier found by Han et al. (2009), it was demonstrated that α_2_ pumps in cardiomyocytes (Stanley et al., 2015) and skeletal muscle (DiFranco et al., 2015) have reduced affinity for extracellular K^+^, allowing them to be readily stimulated by physiological rises in ${\text{K}}_{\text{o}}^{+}$ occurring under exercise. The distinct (cardiomyocytes) and almost exclusive (skeletal muscle) localization of the α_2_- isoform to T-tubules (see DiFranco et al., 2015; Stanley et al., 2015, and references therein), which are highly diffusion-restricted spaces where ${\text{K}}_{\text{o}}^{+}$ may rise to tens of millimolar during muscle activity (DiFranco et al., 2015), further supports the notion that α_2_ provides a safety net for ${\text{K}}_{\text{o}}^{+}$ clearance and Na^+^ extrusion that is only recruited on request.

Should cardiac, skeletal muscle or vasculature deficits accompany the phenotype of ATP1A2 haploinsufficiency? In the heart, the low (about 10–15%) overall proportion of the α_2_- isoform and the presence of potentially compensating α_1_ in T-tubules may alleviate loss of α_2_ activity. Results from transgenic animals seem to depend on the knockout strategy. James et al. investigated a global germline deletion of one copy of α_2_ (heterozygous α_2_(+/−) knockout mice) and observed cardiac hypercontractility as a result of increased Ca^2+^ transients during the contractile cycle, in accordance with the proposed role of α_2_ in cardiac inotropy (James et al., 1999). Later, Rindler et al. generated mice with tissue-targeted knockout of α_2_ that resulted in more than 90% loss of α_2_ exclusively in the cardiovascular system (Rindler et al., 2011) or in the heart (Rindler et al., 2013). These authors found cardiac and vascular contractility unaltered. Similar contrasting results were obtained regarding the effects of α_2_ knockouts in the vasculature, in which the ratio of α_2_ to α_1_ is 30%/70% (Shelly et al., 2004). Whereas heterozygous α_2_(+/−) knockout mice had elevated systolic blood pressure, increased myogenic tone and arterial contractility (Shelly et al., 2004; Rindler et al., 2011), the cardiovascular knockout model showed normal basal blood pressure and vascular contractility suggesting that expression of α_2_ in cardiac myocytes and vascular smooth muscle is not involved in the regulation of basal blood pressure. Possibly, α_2_ in another cell type might be responsible for the hypertension observed in global α_2_(+/−) mice (Rindler et al., 2011). In rare cases, vascular abnormalities coincide with hemiplegic migraine, such as pulmonary arterial hypertension (Montani et al., 2013) and reversible cerebral vasoconstriction (Hermann et al., 2013).

The situation should definitely be different in skeletal muscle, in which α_2_ comprises nearly 90% of total α-subunit content (He et al., 2001) and is almost exclusively present in T-tubules (DiFranco et al., 2015). However, the α_2_- isoform does not set resting ion gradients (He et al., 2001) or the resting potential (Radzyukevich et al., 2004, 2013) in skeletal muscle, the canonical roles of the Na^+^,K^+^-ATPase in most other cell types, due to its profound voltage-dependent inhibition at the about −90 mV resting potential in skeletal muscle and its lower K^+^ affinity compared to α_1_. In accordance, the α_1_- isoform localized to the surface sarcolemma provides up to 75% of the basal Na^+^,K^+^ transport needed to stabilize ion gradients and membrane potential at rest. However, α_1_ operates at the upper edge of its regulatory range for activation by K^+^, and the tremendous transport capacity of α_2_ pumps needs to be recruited during exercise. Skeletal muscle also differs from other excitable tissues because its resting potential is set by ClC chloride channels (Pedersen et al., 2016), as illustrated by the hyperexcitability of skeletal muscle in myotonia congenita, a muscle disease resulting from loss-of-function mutations in the *ClC-1* gene (Koch et al., 1992). Also for skeletal muscle effects, studies on transgenic mice are not fully conclusive. Whereas increased isometric force, hypercontractility and increased fatique was observed in isolated skeletal muscle from heterozygous α_2_(+/−) mice (He et al., 2001), the α_2_(+/−) animals themselves did not fatique faster than wild-type animals (Moseley et al., 2007). Increased fatique was observed in skeletal muscle-targeted α_2_(+/−) knockout animals and muscle preparations derived thereof (Radzyukevich et al., 2013). Of note, a 2.5-fold upregulation of α_1_ was observed in these skeletal muscle α_2_(−/−) knockout animals, and even some presence of α_1_ in T-tubules was observed suggesting that some partial compensation by α_1_ could take place (Radzyukevich et al., 2013). Since the largest effects were observed in the severe skeletal muscle knockout system but not in the global α_2_(+/−) heterozygotes, it seems plausible that the vast capacity of α_2_ to cope with tremendous physiological load increases during physical exercise may keep sufficient reserve at hand that 50% loss in α_2_ may not entail an additional skeletal muscle phenotype in FHM2.

## Methods for experimental assessment of Na^+^,K^+^-ATPase function

### Ouabain survival assays

For the investigation of functional consequences of FHM2 mutations, the so-called ouabain survival assays on HeLa cells are most frequently applied (Bassi et al., 2004; de Vries et al., 2007). The mutations are introduced into an ouabain-resistant ATP1A2 backbone carrying the Q116R/N127D double mutation in the TM1-2 loop, which produces IC_50_ values in the 100 micromolar range (Price and Lingrel, 1988). HeLa cells are either transiently or stably transfected and put under micromolar ouabain stress, which is sufficient to block the endogenous Na^+^ pumps, so that only cells expressing an ATP1A2 mutant construct with sufficient residual activity can survive. The heterozygous state of patients can be mimicked by co-transfecting equal amounts of wild-type and mutant ATP12A2 DNA in order to address possible dominant-negative effects (De Fusco et al., 2003). The cell line for transfection should be carefully chosen as highlighted by the differences observed from ouabain survival assays on COS7 and HeLa cells for the W887R mutation (De Fusco et al., 2003; Leo et al., 2011). Whereas the mutant protein did not confer cell survival in both cell lines, (De Fusco et al., 2003) reported normal cellular distribution and plasma membrane expression in COS7 cells, but (Leo et al., 2011) found drastically reduced protein level and a mainly intracellular distribution in HeLa cells suggesting that COS7 cells might be prone to saturation artifacts, a common threat in transient transfection studies.

### Biochemical assays

Molecular function can be addressed by biochemical assays, which requires recombinant protein production in appropriate expression hosts such as stably transfected mammalian cells (Toustrup-Jensen et al., 2014), or insect cells (e.g., Sf9 derived from the fall armyworm *Spodoptera frugiperda*, Weigand et al., 2014b). Yeast cells (*Pichia pastoris*) have also been used (Cohen et al., 2005), but this host has not yet been employed for the study of FHM2 mutations. Subsequently, the whole set of biochemical techniques can be performed (see Glynn, 1985; Kaplan, 2002, for reviews), such as the “classical” ATPase (Skou, 1957) or phosphorylation assays, in which also the effects of different Na^+^ and K^+^ concentrations, the steady-state phosphoenzyme level, conformational preference, ouabain binding and sensitivity, and vanadate sensitivity can be determined. The maximum phosphorylation levels obtained in the presence of oligomycin are used to measure the concentration of active enzyme sites, which, together with ATP hydrolysis rates, are used to determine the maximum turnover number (Glynn, 1985; Vilsen, 1995), thus yielding a thorough characterization of enzymatic properties.

### Electrophysiological assays: the two-electrode voltage clamp

The cation transport function of the Na^+^,K^+^-ATPase as such can only be investigated in intact cells, which maintain the extra-/intracellular sidedness of substrate access and allow for the application of a membrane (or more general: electrochemical) potential load on cation transport. Na^+^,K^+^ transport and the most significant properties relating to the electrogenicity of the Na^+^ pump are determined by electrophysiology, mostly by applying the two-electrode voltage clamp technique on oocytes from the frog *Xenopus laevis* (Stühmer and Parekh, 1995). This technique allows one to study the dependence of Na^+^,K^+^ pump currents on extracellular [K^+^], [Na^+^], ouabain and voltage, so that the complex interplay between Na^+^ and K^+^ at the externally facing cation binding sites can be analyzed.

**Figure 4:**
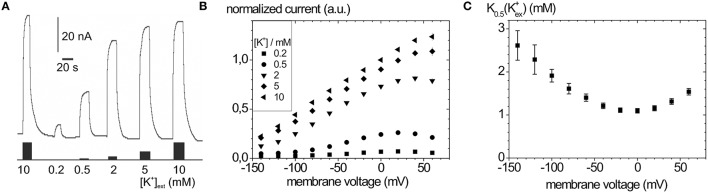
**Stationary Na^**+**^/K^**+**^ pump currents of the Na^**+**^,K^**+**^-ATPase (human ATP1A2) from two-electrode voltage clamp experiments on ***X. laevis*** oocytes. (A)** Pump currents in response to different extracellular [K^+^] at −30 mV holding potential, **(B)** I-V curves for different [K^+^]_ext_ at high [Na^+^]_ext_ (100 mM). **(C)** K_0.5_(K^+^ext) values from fits of a Hill function to the [K^+^] dependent pump current amplitudes at different potentials from **(B)**.

Typical Na^+^, K^+^ pump currents, as can be recorded with the two-electrode voltage clamp on *Xenopus laevis* oocytes, are shown in Figure 4A together with the voltage and extracellular [K^+^] dependence (Figure 4B), from which K_0.5_(K^+^) values for half-maximal pump current stimulation can be determined (Figure 4C), which symbolize the voltage dependence of the enzyme's apparent affinity for extracellular K^+^.

**Figure 5:**
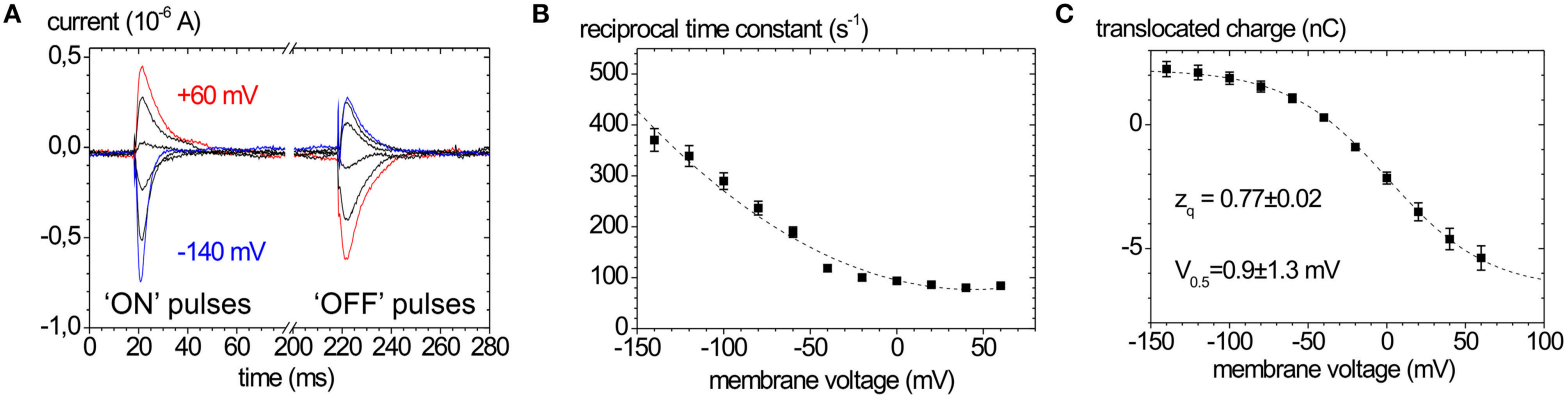
**Properties of ouabain-sensitive transient currents of the Na^**+**^,K^**+**^-ATPase. (A)** Transient currents evoked by pulses from −30 mV to voltages between +60 and −140 mV in −40 mV decrements measured on human ATP1A2 in two-electrode voltage clamp experiments on *X. laevis* oocytes (“ON” currents), and from pulses back to −30 mV (“OFF” pulses). **(B)** Reciprocal time constants from fits of a single exponential function to the current traces in **(A). (C)** *Q(V)* distribution from the improper integrals of the transient current signals (“OFF” pulses) from **(A)** with the parameters obtained from fits of a Boltzmann-type function to the data. The *Q(V)* distribution from “ON” transient currents would be obtained by multiplying the above curve with (−1).

In the absence of extracellular K^+^ and at high extracellular [Na^+^], the enzyme is restricted to the partial reaction sequence underlaid in yellow in Figure 1 that accounts for ouabain-sensitive “transient” currents in response to voltage pulses (Figure 5A). Extracellular release of Na^+^ ions occurs in three distinct steps, from which only the deocclusion and release of the first Na^+^ ion is major electrogenic (Holmgren et al., 2000). This step is rate-limited by the preceding (presumably non-electrogenic) E_1_P → E_2_P conformational change and cation deocclusion, which gives rise to the startling observation that the forward rate constant for Na^+^ release is not voltage-dependent (see the flat progression of rate constants in Figure 5B at positive voltages). However, in the reverse direction, the reverse binding of Na^+^ ions from the extracellular side induced by negative voltage pulses occurs with a strongly voltage-dependent rate constant (see the steeply rising rate constants in Figure 5B at negative voltages). Following release of the first Na^+^ ion, the high-field access channel to the other Na^+^ occlusion sites is restructured such that the exit of the remaining two Na^+^ ions contributes only little to the overall electrogenicity. In terms of the “access channel” model, positive voltage pulses drive Na^+^ ions extracellularly out of the access channel resulting in ouabain-sensitive “transient” currents with positive polarity. Conversely, negative voltage pulses promote the reverse binding of Na^+^ ions to the binding sites in E_2_P and induce negative transient currents (Figure 5A). Due to the electrogenicity of reverse binding of extracellular Na^+^, the occupancy of the Na^+^ binding sites is controlled by [Na^+^]_o_ and voltage. The amount of charge moved in response to a certain voltage step *Q(V)* follows a characteristic, sigmoidal, Boltzmann-type function (see Figure 5C):

(1) $$Q(V)=Q_{\min}+\frac{Q_{\max}-Q_{\min}}{1+e^{-\frac{z_{q}\cdot F}{R\cdot T}(V-V_{0.5})}}$$

which is used to fit the experimentally obtained *Q(V)* curves. Here, *Q*_min_ and *Q*_max_ are the saturation values of *Q(V), F* is the Faraday constant, *R* the molar gas constant, *T* the absolute temperature in K, *V* the membrane voltage, and *z*_*q*_ the slope factor or equivalent charge. This distribution is centered at a half-maximal voltage (*V*_0.5_), at which 50% of Na^+^ binding sites of the pump molecules are occupied. Since the Na^+^ uptake/release steps are kinetically coupled to the E_1_P↔E_2_P conformational transition, 50% of the enzyme molecules are in E_2_P and 50% are in E_1_P at this stage. Thus, the *V*_0.5_ value at a given [Na^+^]_o_ is a characteristic parameter for each Na^+^,K^+^-ATPase isozyme (or mutant), and since *V*_0.5_ shifts with the extracellular [Na^+^], changes in *V*_0.5_ induced by mutations indicate changes in the apparent affinity for ${\text{Na}}_{\text{o}}^{+}$. Positive shifts of *V*_0.5_ indicate an increased apparent Na^+^ affinity and *vice versa*.

The equivalent charge *z*_*q*_ indicates the electrogenicity of the Na^+^ transport step, i.e., which fraction of the transmembrane field is “sensed” by a unitary charge moved or, conversely, which fraction of a charge encounters the full transmembrane field during an elementary charge-moving event.

**Figure 6:**
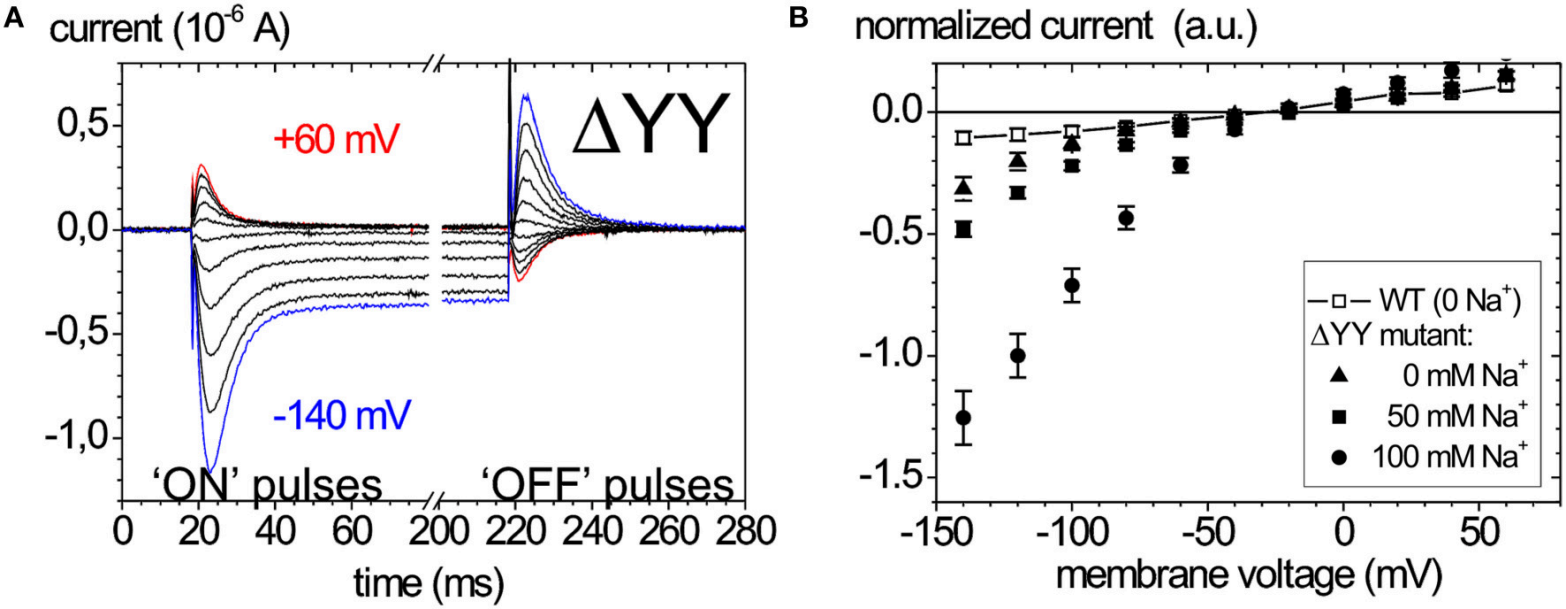
**Leak currents of the Na^**+**^,K^**+**^-ATPase upon mutation of the α-subunit's C-terminus**. **(A)** Ouabain-sensitive transient currents measured at [K^+^]_o_ = 0, [Na^+^]_o_ = 100 mM (pH 7.4) upon pulses from −30 mV to voltages between +60 and −140 mV in −40 mV decrements for mutant ATP1A2-ΔYY in two-electrode voltage clamp experiments on *X. laevis* oocytes (“ON” currents), and from pulses back to −30 mV (“OFF” pulses). **(B)** [Na^+^]_o_ dependence of the current-voltage curves of the steady-state inward leak currents (closed symbols), the leak currents of the WT enzyme at zero [Na^+^] are shown as open squares.

Of late, the ouabain-sensitive leak currents are investigated by electrophysiology as well, since mutations interfering with a C-terminal access pathway for protons drastically affect cation affinities (Morth et al., 2007; Poulsen et al., 2010). Thus, especially C-terminal mutations identified in FHM2 and AHC have been scrutinized for suspicious leak current activity (Poulsen et al., 2010; Li et al., 2015). As an example, the leak currents of the Na^+^ pump that occur upon deletion of the two C-terminal tyrosines of the α-subunit are shown in Figure 6. Figure 6A shows ouabain-sensitive transient currents of the ATP1A2-ΔYY mutant in the absence of extracellular K^+^ and [Na^+^]_o_ = 100mM (Meier et al., 2010). Compared to the transient currents of the WT enzyme (Figure 5A), the “ON” transient currents of the mutant do not decay to zero at negative voltages, but a steady inward current results, which is augmented with increasing [Na^+^]_o_ (Figure 6B). This phenomenon was also observed by Yaragatupalli et al. (2009), Poulsen et al. (2010) and Vedovato and Gadsby (2010).

## ATP1A2 mutations correlated with clinical migraine cases

To date, about 81 *ATP1A2* mutations have been reported in migraine-correlated neurological disease cases in the literature. A complete list is provided in Supplementary Table 1 together with information about the diagnosed diseases, the location of the mutated residues within the structure of the Na^+^,K^+^-ATPase (see Figure 2) and, if available, a brief summary of functional consequences of the mutations. The vast majority (about 60) of the mutations were classified as FHM. Moreover, about 25 mutations were diagnosed in sporadic cases of hemiplegic migraine, SHM, (with overlap in the case of G815R, R908Q, P979L, which were identified in different unrelated pedigrees or individuals) showing that mutations in the *ATP1A2* gene locus substantially account for *de novo* mutations causing hemiplegic migraine. About 10% of *ATP1A2* mutations were identified in migraine with or without aura (MA/MO) indicating that the gene might also be a susceptibility locus for common forms of migraine (Todt et al., 2005). An overlap with epilepsy or seizures has been noted in about 15% of cases. Of note, two ATP1A2 mutations were identified in patients with AHC, such as the I589T mutation reported in an atypical case of AHC (Al-Bulushi et al., 2014), and T378M, which was found in two families, either correlated with FHM (Bassi et al., 2004) or with AHC (Swoboda et al., 2004). Other pathologies associated with *ATP1A2* mutations were sensorineural hearing loss (V191M, Oh et al., 2015), basilar migraine (R548H, Ambrosini et al., 2005), benign familial infantile convulsions (BFIC; R689Q, Vanmolkot et al., 2003), generalized epilepsy with febrile seizures (GEFS+; G874S, Costa et al., 2014), pulmonary arterial hypertension (S940L, Montani et al., 2013) and reversible cerebral vasoconstriction (P979L Hermann et al., 2013).

Within the Na^+^,K^+^-ATPase crystal structure, more than 80% of mutations fall into four spatially distinct clusters, one around the catalytic P domain, one in a central region between P and TM domain, one within the extracellular-facing part of the TM domain, and one around the enzyme's C-terminus (Figure 2), which are all regions of critical importance for function. About 75% of the reported ATP1A2 mutations have been scrutinized for function at different levels of experimental sophistication (Supplementary Table 1).

### Functional studies: mildly and severely deleterious ATP1A2 mutations

Among the ATP1A2 mutations studied so far, some stand out because only mild consequences were observed (Supplementary Table 1). These include mutations at the enzyme's N-terminus, Y9N (SHM) and R51H (MO), which behaved similar to WT in biochemical studies on Sf9 cell membrane preparations (Swarts et al., 2013). A clue for the physiological consequences can be inferred from Song et al. (2006), who showed that ATP1A2 and ATP1A3 share an N-terminal targeting sequence within amino acids 1–90, which is apparently responsible for localization to jS/ER compartments in primary cultured mouse astrocytes. It remains to be clarified whether the N-terminal mutations of ATP1A2 interfere with targeting in order to decide whether or not these mutations are rare missense variants without pathogenic effects. The same accounts for E174K (MO), which was inconspicious in electrophysiological experiments on *Xenopus* oocytes and showed only mildly reduced activity in Sf9 cell preparations (Todt et al., 2005; Swarts et al., 2013), although the mutation inverts the charge of a highly conserved residue in the A domain, which might form a salt bridge with Lys-432 in the N domain important for inter-domain interactions. Very similar observations were reported for the E902K (FHM) mutation, which showed electrophysiological properties like the WT enzyme (Spiller and Friedrich, 2014), but reduced ATPase activity and ouabain binding in Sf9 cell membrane preparations (Swarts et al., 2013). Also for K1003E (SHM+seizures) and R1007W (FHM), only mild consequences were reported from electrophysiology on *Xenopus* oocytes (Pisano et al., 2013; Spiller and Friedrich, 2014), despite the charge inverting/neutralizing effect.

At the other end of the functional spectrum, mutations that lead to a premature stop codon, a frame shift or a deletion/insertion are expected to cause severe functional disruptions. These include K95del (FHM & epilepsy), F305del (SHM), R834X (FHM), del(K935-S940)insI (FHM), S966fs (FHM), L944del (SHM+focal seizures), and Y1009X (SHM), which all classify as familial or *de novo* mutations causing hemiplegic migraine, frequently with severe accompanying symptoms and, as far as functional studies are available, lead to complete loss of function and/or loss of plasma membrane targeting.

**Figure 7:**
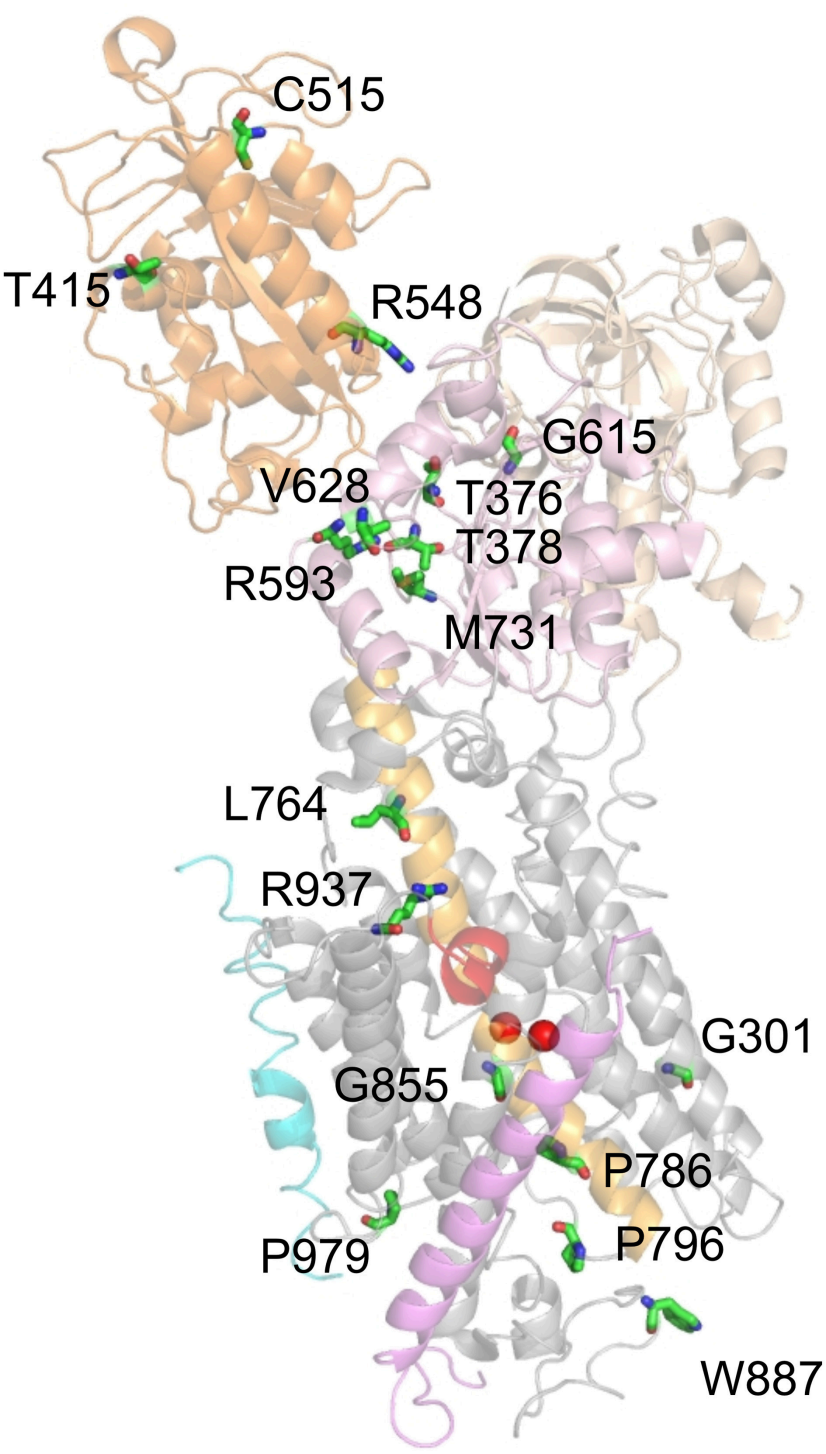
**Location of ATP1A2 mutations with most severe consequences on function**. Mutated amino acids, for which functional consequences are described in the text, are shown in stick representation. Mutant P979L was shown to be fully functional in the *Xenopus* oocyte system, whereas temperature-dependent effects on protein stability and plasma membrane targeting were observed in mammalian cells (Tavraz et al., 2008, 2009).

The remaining mutations, which entail loss or drastic reduction of activity, are depicted in Figure 7. These mutations mostly locate to the P domain and the extracellular TM domain cluster, with only a few others in the N domain (T415M, C515Y, R548H) and two in the central cluster (L764P, R937P). Mutation G301R (identified in two FHM kindreds) showed slightly reduced plasma membrane expression but no pumping activity in the *Xenopus* oocyte system, indicating reduced protein stability and complete loss-of-function (Tavraz et al., 2009). In HeLa cells, however, the mutant showed strongly reduced cell viability in ouabain survival assays, no plasma membrane expression and no protein detectable in Western blots (Santoro et al., 2011). The structure-destabilizing and functional disruption effect can be attributed to the insertion of a bulky, charged side chain within the center of the block of TM helices close to one of the common cation binding sites.

T376M (FHM) and T378N (FHM, AHC) affect two threonines in the ^373^S**D**KTGTLT^380^ motif around the intermediately phosphorylated Asp-374. Although the mutant proteins were expressed similar to the WT enzyme in *Xenopus* oocytes (T376M, Tavraz et al., 2008) and HeLa cells (T378N, Bassi et al., 2004), no pump activity or cell survival was observed. T415M (FHM) and C515Y (MA) affect two highly conserved residues at the periphery of the N domain, for which effects on function are difficult to infer. Nevertheless, the T415M mutant did not confer cell viability in ouabain survival assays despite normal protein level (Vanmolkot et al., 2007), and the C515Y mutation entailed strongly reduced pump currents and ATPase activity in *Xenopus* oocytes (Todt et al., 2005). Inter-domain interactions are also a critical concern for the R584H (basilar migraine/MA) and R548C (FHM) mutations, since Arg-548 likely forms a salt bridge with Glu-221 that stabilizes the interaction between the A and N domains in the E_2_P conformation, but probably forms another salt bridge to the β-phosphate of ATP. Although ATP affinity was not changed for mutant enzymes prepared from Sf9 cells, strongly reduced ATPase activities were observed (Swarts et al., 2013) indicating that the structure-coordinating effect of the salt bridge might be critical.

Mutations R593W and V628M were both identified in FHM and showed strongly reduced cell viability in ouabain survival assays with normal protein level (Vanmolkot et al., 2006a), and R593W exhibited strongly reduced ATPase activity and reduced phosphoenzyme level in COS-1 cell membranes (Schack et al., 2012). Arg-593 is located at the border of the P domain, directly after two prolines that form the hinge between the P and N domains. The residue is within hydrogen bonding distance of the backbone carbonyls of Gly-377 and Thr-378, which are located in the same loop as Asp-374 and Thr-376 indicating that the steric clash resulting from insertion of the tryptophan might disturb phosphorylation (Vanmolkot et al., 2006a; Schack et al., 2012). Val-628 resides in the P domain and is located at the border of the short, conserved P3 helix that together with the seven-stranded parallel β-sheet forms the typical Rossmann fold implicated in nucleotide binding suggesting an impact on catalytic activity (Schack et al., 2012). The G615R mutation, which was identified in FHM with particularly severe accompanying symptoms and in a patient with SHM, affects the glycine in the critical ^612^MVTGD^616^ structure motif in the P domain, which rationalizes that the mutation has been found to be deleterious for cell viability (Vanmolkot et al., 2006b).

The M731T mutation was functionally assessed by several groups (Capendeguy and Horisberger, 2004; Segall et al., 2005; Schack et al., 2012), with the most stringent study carried out on the human ATP1A2 enzyme (Schack et al., 2012), whereas the other two studied rat ATP1A2 (Segall et al., 2005) or ATP1A1 from *Bufo marinus* (Capendeguy and Horisberger, 2004). All studies converged on the notion of strongly reduced ATPase activity or loss of pump function. For efficient phosphorylation, the loop between β-sheet 6 and helix P7 of the Rossmann fold, in which Met-731 is located, has to be strained upon Mg^2+^ binding. Thus, the role of Met-731 may be to reduce the mobility of this loop to ensure the strain. Met-731 is also flanked by Arg-593 (see above), and the mutation might prevent proper bending of the P domain (Schack et al., 2012).

Mutations L764P and W887R were identified in the first report on FHM2 (De Fusco et al., 2003). L764P inserts a structure-breaking proline into the central helix TM5, which connects the phosphorylation site to the cation-binding pocket. The P786L (SHM) mutation also affects a residue on the extracellularly-oriented part of the TM5 helix. In accordance with the critical role of TM5, all studies investigating the effects of the TM5 mutations showed severe or complete loss of catalytic activity (Koenderink et al., 2005; Swarts et al., 2013) or cell survival (De Fusco et al., 2003; de Vries et al., 2007). The ^796^P796S^799^ (FHM) mutation, which affects a proline within the conserved PLPL turn connecting TM5 and TM6, also did not support cell survival (Castro et al., 2008) or ATPase activity in Sf9 cell membranes (Weigand et al., 2014b).

The W887R mutant also did not confer cell survival (De Fusco et al., 2003), showed no pump currents (Capendeguy and Horisberger, 2004; Koenderink et al., 2005) and strongly reduced Rb^+^ uptake, despite normal plasma membrane expression in *Xenopus* oocytes (Koenderink et al., 2005). From the structure, it is difficult to rationalize a loss of functional activity by this mutation. Trp-887 is located within the TM7-8 loop important for interaction with the β-subunit, but is not directly involved (Nyblom et al., 2013). If the interaction with the β-subunit were disrupted, a defect in membrane insertion and protein folding accompanying biosynthesis in the ER could be expected, with consequently reduced plasma membrane targeting, but this was not observed in the expression systems studied. Furthermore, the defective ouabain binding of the W887R mutant (Koenderink et al., 2005) is puzzling since Trp-887 was not implicated in ouabain binding. However, it was found in a mouse model for FHM2 that the mutant ATP1A2 protein was hardly detectable in the brain of homozygous ATP1A2(W887R/W887R) mutants and strongly reduced in ATP1A2(+/W887R) heterozygous mutants (Leo et al., 2011). In transfected HeLa cells, these authors also found profound protein loss, likely as the consequence of endoplasmic reticulum retention and subsequent proteasomal degradation. This finding contrasts with observations from the aforementioned study on transfected COS7 cells (De Fusco et al., 2003) indicating that it is important to avoid saturation effects in cell line used for transfection (Leo et al., 2011).

Mutation G855R (FHM+febrile seizures) did not confer cell survival (de Vries et al., 2009) or pump currents in the *Xenopus* oocyte system (Spiller and Friedrich, 2014), which in the latter system coincided with strongly reduced plasma membrane protein level. Gly-855 resides within TM7, a helix forming multiple contacts to the TM of the β-subunit (Nyblom et al., 2013), but Gly-855 is not directly involved in helix-helix contacts. However, due to the close packing of the helices, the introduction of a bulky side chain can indirectly affect the interaction with the β-subunit, which appears likely since the defective plasma membrane expression suggests improper folding and premature degradation.

Mutation R937P (FHM) did not show pump currents or Rb^+^ uptake in an initial study on *Xenopus* oocytes (Tavraz et al., 2008), and no ATPase activity in Sf9 cell membranes (Weigand et al., 2014b). However, based on the knowledge about the involvement of Arg-937 in coordinating the α-subunit's C-terminus, its link to Na^+^ binding site III and the reported effects on Na^+^ affinity (Toustrup-Jensen et al., 2009), it could be shown by electrophysiology that the mutation caused enhanced proton “leak” currents and a drastic negative shift of the *Q(V)* curve from transient currents (Poulsen et al., 2010), in line with a drastically reduced apparent affinity for extracellular Na^+^. In effect, the R937P mutant still retains some essential properties of the Na^+^ pump, albeit with severely changed voltage dependence and enhanced proton leak, but the properties of the mutant's Na^+^, K^+^ pump currents have still to be elucidated. R937P is so far the only FHM2 mutation that shows increased proton leak currents. Work by the Artigas and Gadsby labs showed that pump-mediated proton current is an intrinsic property of Na^+^,K^+^-ATPase (Mitchell et al., 2014; Vedovato and Gadsby, 2014) at physiological K^+^ and Na^+^ concentrations and resting potentials suggesting that this “hybrid” transporter function may well have been exploited by nature for some physiological purpose. It still remains to be established whether Na^+^,K^+^-ATPase-mediated proton uptake plays any physiological or pathological role (see Section Functional Properties of the Na^+^,K^+^-ATPase).

### Mutation P979L entails temperature-dependent protein instability and mistargeting

P979L is a particularly interesting mutation, which was reported to cause FHM2 in one of the earliest accounts on the genetic background of the disease (Jurkat-Rott et al., 2004), and, later, also in a case of SHM (Hermann et al., 2013). Comorbidities were serious, since the first report listed particularly severe attacks accompanied by recurrent coma and tonic-clonic seizures, whereas the SHM patient suffered from prolonged aura phase and severe reversible cerebral vasoconstriction. Within the crystal structure, Pro-979 is located within the TM9-10 loop and the protein backbone kink induced by the proline may be important for protein folding. However, when assayed by electrophysiology in *Xenopus* oocytes, all functional parameters were identical to those of the WT enzyme, with normal total and plasma membrane protein levels (Tavraz et al., 2008). However, in surface biotinylation assays on transfected HEK293FT cells, it was later found that the amount of P979L protein was strongly reduced, when the cells were incubated at 37°C, but not upon cell incubation at 28°C (Tavraz et al., 2009). This discrepancy highlights the influence of the model cell system used. *Xenopus* oocytes are kept at 18–20°C, a temperature range, which is advantageous for protein folding, whereas mammalian cells are grown at 37°C, where protein instability might lead to misfolding and degradation. The situation is reminiscent of observations on the epithelial CFTR Cl^−^ channel mutated in cystic fibrosis (CF). The most common CF mutation, the ΔF508 deletion, leads to rapid degradation of the protein before exiting the ER in mammalian cells (kept at 37°C), while the mutant protein could be functionally expressed in *Xenopus* oocytes. It could be shown that this effect is temperature-sensitive since protein degradation can be rescued at permissive temperature (Denning et al., 1992).

## Summary and perspectives

The detailed atomic-scale understanding of ion transport and catalysis of the Na^+^,K^+^-ATPase provided by the wealth of structural data together with the multi-modal efforts of numerous experimentalists have provided a rather stringent concept of the effects of migraine-associated ATP1A2 mutations on molecular function. While the spectrum of functional disruptions matches the complexity of the Na^+^ pump's inner workings and the enzyme's even more complex integration into cellular signaling networks, the notion emerges that loss or change of any kind of functional parameter, including the seemingly subtle changes in voltage dependence could be of pathophysiological relevance. This is because for any of these alterations, pathophysiological conditions are conceivable that render these changes critical for controlling the excitability of electrically excitable tissues. Two seemingly self-evident but oversimplifying conclusions should be avoided: First, changes in Na^+^ pump function brought about by disease-related mutations do not converge on a simple loss-of-function concept. While this is certainly true for mutations abolishing Na^+^,K^+^ pumping, a change in voltage dependence may entail loss-of-function in a particular voltage range, but gain-of-function in another. Furthermore, given the controversy about whether proton leak currents are part of the Na^+^ pump's physiological spectrum of functions, both, reduced proton leaks (loss-of-function, as discussed for the E815K mutation in ATP1A3 in AHC) as well as increased proton leaks (gain-of-function, as discussed for somatic ATP1A1 mutations in APA) could be causative for one or the other pathophysiological state. Second, the CSD phenomenon is not simply the consequence of either cortical hypo- or hyperexcitability. Rather, it emerges that the reduced ability to dynamically maintain the *cortical excitatory/inhibitory balance* and the failure to prevent excessive increases in cortical excitation mechanistically explain the abnormal sensory processing in migraineurs.

While it is commonly accepted that functional changes in FHM-related genes including those in *ATP1A2* converge on CSD as the neurophysiological correlate of migraine aura, the link to the most disabling condition of the disease, the throbbing migraine pain, is still a matter of debate. Currently, the view emerges that CSD can cause sustained activation of meningeal nociceptors and central trigeminovascular neurons to initiate the headache mechanisms in a process termed sterile meningeal inflammation. Moreover, the physiological control parameters discriminating between the propagation speeds of rapid (epileptic seizures) or slow (CSD) waves of cortical hyperexcitation need to be investigated in more detail to identify critical network parameters for or by *in silico* modeling of excitable biological matter. In this context, knowing the parameters of dysfunction of mutated ion pumps and channels of the CNS may help to identify the elements that count. The remarkable progress achieved by computational neuroscientists who have just recently put forward the idea that cell volume regulation is critical for determining whether an excitable tissue may evolve into seizures or spreading depression, provides novel hypotheses to be tested by experiment. This should encourage synergistic, cross-disciplinary collaborations between researchers studying excitable matter on the clinical, *in vivo, in vitro*, and *in silico* level.

In order to classify an ATP1A2 mutation as causative for a disease, at least the simplest test should be performed, an ouabain survival assay in mammalian cells. For the assessment of enzyme catalysis, biochemical studies on protein preparations from mammalian cell lines or Sf9 cells provide exhaustive tools of characterization. However, elucidation of the most essential physiological function, electrogenic Na^+^,K^+^ transport, requires dedicated electrophysiological assays on intact cells. For this purpose, the *Xenopus* oocyte expression system is a versatile tool, since it permits electrophysiology as well as cation uptake assays, and some means of fundamental protein biochemistry. Whereas electrophysiology on oocytes is indispensible for researchers interested in understanding Na^+^ pump function in molecular detail, the *Xenopus* system is less physiological when it comes to effects on protein expression, stability or targeting, as the example of the P979L mutant and others noted in this work have shown. Thus, as a rule of thumb, the observed functional consequences should ideally be cross-checked in at least two different cell systems in order to avoid both false-positive and false-negative conclusions.

## Author contributions

TF and NT performed literature searches, TF prepared figures and all authors wrote the manuscript.

### Conflict of interest statement

The authors declare that the research was conducted in the absence of any commercial or financial relationships that could be construed as a potential conflict of interest.
