# Supplementary material for: ATP1A2 Mutations in Migraine: Seeing through the Facets of an Ion Pump onto the Neurobiology of Disease
Source: Front Physiol. 2016 Jun 21;7:239. doi: 10.3389/fphys.2016.00239 (PMC4914835; doi:10.3389/fphys.2016.00239)
Supplement: Supplementary file 1 [file Table1.PDF]

ATP1A2 mutations in migraine: Seeing through the facets of an ion pump onto the neurobiology of disease

Thomas Friedrich*, Neslihan N. Tavraz and Cornelia Junghans

Technical University of Berlin, Institute of Chemistry PC 14, Straße des 17. Juni 135, D-10623 Berlin, Germany

Supplementary Information

Supplementary Table

ATP1A2 mutations identified in migraine, as published in the literature until May 2016. For each mutation the characteristic clinical phenotype is provided with the respective reference (FHM - familial hemiplegic migraine, SHM - sporadic hemiplegic migraine, MA/MO - migraine with/without aura, BM - basilar migraine), the location of the mutation position within the Na^+,K^+-ATPase structure, reference(s) reporting functional tests (as available) and a summary of the findings obtained from these studies. The listed references were identified with searches for the keyword combination "[migraine] and ([ATP1A2] or [ATPase])" in title or abstract.

ATP1A2 allelic variant	mutation type	phenotype	localisation in 3D structure	reference (genetics)	reference (functional test)	effect(s) on function (if any)
**Y9N**	missense mutation	SHM	A domain	(Gallanti et al., 2011) (Thomsen et al., 2008) (Tonelli et al., 2007)	(Swarts et al., 2013)	Sf9 cells: normal protein expression, ouabain binding and ouabain affinity; apparent K^+ and Na^+ affinity, ATP affinity and turnover as WT in ATPase assay
**K35del**	deletion	FHM & epilepsy	A domain	(Riant et al., 2010)		
**R51H**	missense mutation	MO	A domain	(Castro et al., 2008a)	(Swarts et al., 2013)	Sf9 cells: normal protein expression, ouabain binding and ouabain affinity; apparent K^+ and Na^+ affinity, ATP affinity and turnover as WT in ATPase assay
**R65W**	missense mutation	FHM	A domain	(Gallanti et al., 2011) (Tonelli et al., 2007)		
**E120A**	missense mutation	SHM	TM1-2 loop, ouabain binding region	(de Vries et al., 2007)	(de Vries et al., 2007)	HeLa cells: reduction of survival under ouabain challenge, normal protein level
**V138A**	missense mutation	FHM	TM2	(Thomsen et al., 2007)	(Schack et al., 2012)	COS-1 cells (membrane fractions): ATPase assays: decreased ouabain affinity and turnover rate, apparent K^+ affinity as WT, increased ATP affinity, reduced vanadate sensitivity; Phosphorylation assays: reduced Na^+ affinity and phosphoenzyme level, reduced V_{max}
**E174K**	missense mutation	MO	A domain	(Todt et al., 2005)	(Todt et al., 2005) (Swarts et al., 2013)	*X. laevis* oocytes: ATPase activity identical to WT, electrophysiology: pump currents identical to WT, normal overall protein level<br>Sf9 cells: normal protein expression, ouabain binding and ouabain affinity; reduced ATPase activity with similar ATP affinity and apparent K^+ and Na^+ affinity, ATP affinity, reduced turnover in ATPase assay compared to WT

**V191M**	missense mutation	migraine & sensorineural hearing loss	A domain	(Oh et al., 2015)	(Oh et al., 2015)	Protein purified from Sf9 cells: no reduction in ouabain binding, no change in apparent Na^+ and K^+ affinity of ATPase activity<br>Electrophysiology on *X. laevis* oocytes: Pump currents, K^+ and voltage dependence of pump currents and voltage dependence of ouabain-sensitive Na^+/Na^+ exchange currents identical to WT
**R202Q**	missense mutation	FHM	A domain	(Thomsen et al., 2007)	(Schack et al., 2012)	COS-1 cells (membrane fractions): ATPase assays: ouabain affinity as WT, slightly reduced turnover rate, apparent K^+ affinity as WT, slightly increased ATP affinity, reduced vanadate sensitivity; Phosphorylation assays: Na^+ affinity and phosphoenzyme level as WT
**S220L**	missense mutation	FHM	A domain, TGE**S** motif	(Roth et al., 2014)		
**T263M**	missense mutation	FHM	A domain	(Riant et al., 2005)	(Tavraz et al., 2008)<br>(Schack et al., 2012)	*X. laevis* oocytes: No pump currents, strongly reduced Rb^+ uptake, normal overall and plasma membrane protein level<br>COS-1 cells (membrane fractions): ATPase assays: strongly decreased ouabain affinity, reduced turnover rate, increased apparent K^+ affinity, increased ATP affinity, reduced vanadate sensitivity; Phosphorylation assays: slightly reduced Na^+ affinity and phosphoenzyme level
**I286T**	missense mutation	FHM (compound heterozygous with T415M)	TM3	(Vanmolkot et al., 2007)	(Vanmolkot et al., 2007)	HeLa cells: reduction of survival under ouabain challenge, normal protein level

**G301R**	missense mutation	FHM, seizures, cerebellar signs	TM3	(Spadaro et al., 2004)<br>(Santoro et al., 2011)	(Santoro et al., 2011)<br>(Tavraz et al., 2009)	HeLa cells: reduction of survival under ouabain challenge, no plasma membrane expression (c-myc epitope reactivity), no protein detectable in Western blot<br>*X. laevis* oocytes: No pump current, strongly reduced Rb^+ uptake, total and plasma membrane protein level as WT
**F305del**	deletion	SHM	TM3	(Riant et al., 2010)		
**V338A**	missense mutation	SHM	TM4-5 loop	(Riant et al., 2010)		
**c.1025T>C L342P**	missense mutation	FHM	P domain	(Asghar et al., 2012)		
**T345A**	missense mutation	FHM	P domain	(Kaunisto et al., 2004)	(Segall et al., 2004)<br>(Weigand et al., 2014)<br>(Schack et al., 2012)	HeLa cells: normal expression and cell growth under ouabain challenge, normal catalytic turnover (V_{max}), reduced apparent K^+, ATP affinity and vanadate sensitivity (ATPase activity tests), reduced affinity in $^{86}Rb^+$ uptake assay; tests performed on rat ATP1A2<br>Sf9 cells: normal protein expression and slightly increased ouabain binding compared to WT, slightly decreased Na^+ and K^+ affinity in ATPase assay<br>COS-1 cells (membrane fractions): ATPase assays: decreased ouabain affinity and turnover rate, decreased apparent K^+ affinity as WT, increased ATP affinity, decreased vanadate sensitivity; Phosphorylation assays: reduced Na^+ affinity and phosphoenzyme level, strongly reduced V_{max}
**V362E**	missense mutation	FHM	P domain	(Castro et al., 2008b)	(Castro et al., 2008b)	HeLa cells: strongly reduced cell survival under ouabain challenge, normal protein level
**T364M**	missense mutation	SHM, FHM, aphasia	P domain	(Riant et al., 2005)<br>(Castro et al., 2007)<br>(Toldo et al., 2010)		
**T368K**	missense mutation	SHM	TM4-5 loop	(Riant et al., 2010)		

**T376M**	missense mutation	FHM	P domain	(Riant et al., 2005) (Castro et al., 2007)	(Tavraz et al., 2008)	*X. laevis* oocytes: No pump currents, strongly reduced Rb^+ uptake, normal overall and plasma membrane protein level
**T378N**	missense mutation	FHM AHC	P domain	(Bassi et al., 2004) (Swoboda et al., 2004)	(Bassi et al., 2004)	HeLa cells: strongly reduced cell survival under ouabain challenge, normal *in vitro* protein translation and normal cellular expression pattern (c-myc reactivity), protein detected normally in microsomal and cytosolic membrane fractions
**R383H**	missense mutation	SHM	N domain	(Jurkat-Rott et al., 2004)	(Tavraz et al., 2008)	*X. laevis* oocytes: Reduced pump currents, reduced turnover rate, but apparent K^+ affinity from pump currents as WT, normal overall and plasma membrane protein level; voltage dependence of ouabain-sensitive Na^+/Na^+ exchange currents positively shifted compared to WT (↔increased apparent affinity for extracellular Na^+), kinetics of Na^+/Na^+ exchange currents similar to WT
**T415M**	missense mutation	FHM (compound heterozygous with I286T)	N domain	(Vanmolkot et al., 2007)	(Vanmolkot et al., 2007)	HeLa cells: no survival under ouabain challenge, normal protein level
**E492K**	missense mutation	SHM	N domain	(de Vries et al., 2007)	(de Vries et al., 2007)	HeLa cells: reduction of survival under ouabain challenge, normal protein level
**R510S**	polymorphism	?	N domain	(Thomsen et al., 2007)		
**C515Y**	missense mutation	MA	N domain	(Todt et al., 2005)	(Todt et al., 2005)	*X. laevis* oocytes: ATPase activity strongly reduced, normal overall protein level; electrophysiology: strongly reduced pump currents
**R548H**	missense mutation	BM/MA	N domain	(Ambrosini et al., 2005)	(Swarts et al., 2013)	Sf9 cells: normal protein expression, slightly reduced ouabain binding with normal ouabain affinity; increased apparent K^+ and decreased apparent Na^+ affinity, strongly reduced ATPase activity with similar ATP affinity and strongly reduced turnover in ATPase assay compared to WT

**R548C**	missense mutation	FHM	P domain	(Lebas et al., 2008)	(Swarts et al., 2013)	Sf9 cells: normal protein expression, similar ouabain binding and ouabain affinity; increased apparent K^+ and decreased apparent Na^+ affinity, strongly reduced ATPase activity with similar ATP affinity and strongly reduced turnover in ATPase assay compared to WT
**I589T**	missense mutation	Atypical AHC & generalized seizures	P domain	(Al-Bulushi et al., 2014)		
**R593W**	missense mutation	FHM	P domain	(Vanmolkot et al., 2006a)	(Vanmolkot et al., 2006a)<br>(Schack et al., 2012)	HeLa cells: strongly reduced survival upon ouabain challenge, normal protein level<br>COS-1 cells (membrane fractions): ATPase assays: strongly decreased ouabain affinity and turnover rate, increased apparent K^+ affinity, increased ATP affinity, strongly reduced vanadate sensitivity; Phosphorylation assays: reduced Na^+ affinity, strongly reduced phosphoenzyme level and V_{max}
**V600A**	missense mutation	FHM	TM4-5 loop	(De Cunto et al., 2012)		
**R604P**	missense mutation	SHM	TM4-5 loop	(Riant et al., 2010)		
**A606T**	missense mutation	FHM,<br>FHM &transient learning disorder	P domain	(Riant et al., 2005)<br>(Jen et al., 2007)<br>(Carreño et al., 2013)<br>(Podestà et al., 2011)	(Jen et al., 2007)<br>(Tavraz et al., 2008)	HeLa cells: strongly reduced survival upon ouabain challenge<br>*X. laevis* oocytes: reduced pump currents, reduced turnover rate, normal overall and plasma membrane protein level, reduced apparent affinity for K^+ of pump currents at all voltages tested, strong positive shift in voltage dependence of ouabain-sensitive Na^+/Na^+ exchange currents shifted compared to WT (↔decreased apparent affinity for extracellular Na^+), rate constants from Na^+/Na^+ exchange currents increased at negative potentials compared to WT

**G615R**	missense mutation	FHM<br>SHM	P domain	(Vanmolkot et al., 2006b)<br>(Riant et al., 2010)	(Vanmolkot et al., 2006b)	HeLa cells: strongly reduced survival upon ouabain challenge, normal protein level
**G615E**	missense mutation	SHM	P domain	(Riant et al., 2010)		
**V628M**	missense mutation	FHM	P domain	(Vanmolkot et al., 2006a)	(Vanmolkot et al., 2006a)<br>(Schack et al., 2012)	HeLa cells: strongly reduced survival upon ouabain challenge, normal protein level<br>COS-1 cells (membrane fractions): ATPase assays: decreased ouabain affinity, strongly reduced turnover rate, increased apparent K^+ affinity, slightly increased ATP affinity, strongly reduced vanadate sensitivity; Phosphorylation assays: reduced Na^+ affinity, strongly reduced phosphoenzyme level and V_{max}
**R689Q**	missense mutation	FHM & BFIC	P domain	(Vanmolkot et al., 2003)	(Segall et al., 2005)<br>(Capendeguy and Horisberger, 2004)	HeLa cells: reduced cell survival under ouabain challenge; reduced vanadate sensitivity, unchanged apparent ATP affinity, reduced catalytic turnover (V_{max} from ATPase activity vs. oligomycin-stabilized phosphoenzyme level), normal apparent Na^+ and reduced apparent K^+ affinity in ATPase assays, but 5.6-fold increased protein level compared to WT; reduced apparent K^+ affinity in $^{86}Rb^+$ uptake assay; tests performed on rat ATP1A2<br>*X. laevis* oocytes: protein expression similar to WT in pulse chase labeling experiment, reduced ouabain binding; homologous *Bufo marinus* α1 mutant: reduced palytoxin-induced current, no pump current

**E700K**	missense mutation	FHM	P domain	(Pierelli et al., 2006)	(Swarts et al., 2013) (Schack et al., 2012)	Sf9 cells: normal protein expression, strongly reduced ouabain binding with normal ouabain affinity; strongly reduced turnover in ATPase assay compared to WT<br>COS-1 cells (membrane fractions): ATPase assays: increased ouabain affinity, reduced turnover rate, decreased apparent K^+ affinity, increased ATP affinity, strongly reduced vanadate sensitivity; Phosphorylation assays: reduced Na^+ affinity and slightly increased phosphoenzyme level, reduced V_{max}
**C702Y**	missense mutation	MA/MO & epilepsy	P domain	(Deprez et al., 2008)	(Swarts et al., 2013)	Sf9 cells: normal protein expression, reduced ouabain binding with slightly increased ouabain affinity; apparent K^+ and Na^+ affinity as WT, reduced ATPase activity with decreased ATP affinity and reduced turnover in ATPase assay compared to WT
**V711L**	missense mutation	FHM	TM4-5	(Riant et al., 2010)		
**G715R**	missense mutation	SHM	P domain	(De Sanctis et al., 2011)		
**N717K**	missense mutation	SHM	P domain	(Jen et al., 2007)	(Jen et al., 2007)	HeLa cells: strongly reduced survival under ouabain challenge
**D718N**	missense mutation	FHM	P domain	(Jurkat-Rott et al., 2004)		
**A721T**	missense mutation	SHM + MO, speech deficits	TM4-5	(Riant et al., 2010)		

**M731T**	missense mutation	FHM, BFIC, psychotic aura	P domain	(Vanmolkot et al., 2003)<br>(Castro et al., 2007)<br>(Barros et al., 2012)	(Segall et al., 2004)<br>(Segall et al., 2005)<br>(Capendeguy and Horisberger, 2004)<br>(Schack et al., 2012)	HeLa cells: normal cell survival under ouabain challenge; strongly reduced vanadate sensitivity, reduced apparent ATP affinity, strongly reduced catalytic turnover (V_{max} from ATPase activity vs. oligomycin- stabilized phosphoenzyme level), normal apparent Na^+ and reduced apparent K^+ affinity in ATPase assays, but 5.6-fold increased protein level compared to WT; reduced apparent K^+ affinity in $^{86}Rb^+$ uptake assay; tests performed on rat ATP1A2<br>*X. laevis* oocytes: protein expression similar to WT in pulse chase labeling experiment, reduced ouabain binding; homologous *Bufo marinus* α1 mutant: palytoxin-induced current as WT, no pump current<br>COS-1 cells (membrane fractions): ATPase assays: strongly reduced ouabain affinity and turnover rate, increased apparent K^+ affinity, increased ATP affinity, strongly reduced vanadate sensitivity; Phosphorylation assays: strongly increased Na^+ affinity, reduced phosphoenzyme level, strongly reduced V_{max}
**M745I**	missense mutation	SHM	P domain	(Thomsen et al., 2008)		
**c.2273G>C G758A**	missense mutation	SHM	TM5	(Aceves et al., 2013)		
**R763H**	missense mutation	FHM	TM5	(Jurkat-Rott et al., 2004)	(Tavraz et al., 2008)	*X. laevis* oocytes: reduced pump currents, reduced turnover rate, normal overall and plasma membrane protein level, increased apparent affinity for K^+ of pump currents at all voltages tested, voltage dependence and kinetics of ouabain-sensitive Na^+/Na^+ exchange currents similar to WT
**R763C**	missense mutation	FHM	TM5	(Thomsen et al., 2007)		

**L764P**	missense mutation	FHM	TM5	(De Fusco et al., 2003)	(De Fusco et al., 2003)<br>(Capendeguy and Horisberger, 2004)<br>(Koenderink et al., 2005)	COS7 cells: no cell survival under ouabain challenge, normal *in vitro* protein synthesis, physiological location of and mutant protein in the membrane fraction<br>*X. laevis* oocytes: protein expression similar to WT in pulse chase labeling experiment, no ouabain binding; homologous *Bufo marinus* α1 mutant: reduced palytoxin-induced current, no pump current<br>*X. laevis* oocytes: Plasma membrane expression in similar to WT, ouabain binding strongly reduced, ATPase activity strongly reduced (oocyte total membranes), Rb^+ uptake strongly reduced; electrophysiology: strongly reduced pump currents
**Y775C**	missense mutation	SHM + epilepsia (febrile seizures)	TM5	(Riant et al., 2010)		
**P786L**	missense mutation	SHM	TM5	(de Vries et al., 2007)	(de Vries et al., 2007)<br>(Swarts et al., 2013)	HeLa cells: no cell survival under ouabain challenge, normal protein level<br>Sf9 cells: slightly reduced protein expression, no ouabain binding; no ATPase activity
**P796R**	missense mutation	FHM	TM5-6 loop	(Jurkat-Rott et al., 2004)		
**P796S**	missense mutation	FHM	TM5-6 loop	(Castro et al., 2008b)	(Castro et al., 2008b)<br>(Weigand et al., 2014)	HeLa cells: strongly reduced cell survival under ouabain challenge, normal protein level<br>Sf9 cells: normal protein expression, no ouabain binding, no ATPase activity
**E825K**	missense mutation	FHM & febrile seizures	TM6-7 loop	(Carreño et al., 2013)	(Carreño et al., 2013)	HeLa cells: strongly reduced cell survival under ouabain challenge, reduced protein level

**M829R**	missense mutation	FHM	TM6-7 loop	(Riant et al., 2005)	(Tavraz et al., 2008) (Weigand et al., 2014)	*X. laevis* oocytes: slightly reduced pump currents, normal turnover rate, normal overall and plasma membrane protein level, apparent affinity for K^+ of pump currents similar to WT, negative shift in voltage dependence of ouabain-sensitive Na^+/Na^+ exchange currents shifted compared to WT (↔decreased apparent affinity for extracellular Na^+), rate constants from Na^+/Na^+ exchange currents increased at all potentials tested compared to WT<br>Sf9 cells: normal protein expression, no ouabain binding, no ATPase activity
**R834Q**	missense mutation	FHM	TM6-7 loop	(Riant et al., 2005)	(Tavraz et al., 2008) (Weigand et al., 2014) (Schack et al., 2012)	*X. laevis* oocytes: reduced pump currents, strongly reduced turnover, normal overall and plasma membrane protein level, apparent affinity for K^+ of pump currents as WT, positive shift in voltage dependence of ouabain-sensitive Na^+/Na^+ exchange currents shifted compared to WT (↔increased apparent affinity for extracellular Na^+), rate constants from Na^+/Na^+ exchange currents increased at all potentials tested compared to WT<br>Sf9 cells: normal protein expression, strongly reduced ouabain binding, strongly reduced ATPase activity, increased apparent K^+ and decreased apparent Na^+ affinity in ATPase assay<br>COS-1 cells (membrane fractions): ATPase assays: strongly decreased ouabain affinity and turnover rate, increased apparent K^+ affinity, increased ATP affinity, strongly reduced vanadate sensitivity; Phosphorylation assays: strongly reduced Na^+ affinity, phosphoenzyme level and V_{max}
**R834X**	non-sense mutation	FHM	TM6-7 loop	(de Vries et al., 2007)	(de Vries et al., 2007) (Weigand et al., 2014)	HeLa cells: no cell survival under ouabain challenge, normal protein level<br>Sf9 cells: normal protein expression, no ouabain binding, no ATPase activity

**G855R**	missense mutation	FHM & febrile seizures	TM7	(de Vries et al., 2009)	(de Vries et al., 2009)<br>(Spiller and Friedrich, 2014)	HeLa cells: no cell survival under ouabain challenge, normal protein level<br>*X. laevis* oocytes: no pump currents, normal overall protein expression but strongly reduced plasma membrane protein
**G855V**	missense mutation	SHM	TM7	(Riant et al., 2010)		
**G874S**	missense mutation	FHM, epilepsy (GEFS+)	TM7-8 loop	(Costa et al., 2014)		
**R879Q**	missense mutation	SHM	TM7-8 loop	(Thomsen et al., 2008)	(Swarts et al., 2013)	Sf9 cells: normal protein expression, reduced ouabain binding and ouabain affinity; apparent K^+ and Na^+ affinity as WT, reduced ATPase activity with decreased ATP affinity and normal turnover in ATPase assay compared to WT
**R879W**	missense mutation	SHM	TM7-8 loop	(Thomsen et al., 2008)	(Weigand et al., 2014)	Sf9 cells: normal protein expression, reduced ouabain binding, reduced ATPase activity, sligntly decreased apparent Na^+ affinity and slightly increased apparent K^+ affinity in ATPase assay
**I883L**	polymorphism	?	TM7-8 loop	(Jurkat-Rott et al., 2004)	(Tavraz et al., 2009)	HEK293FT cells: plasma membrane protein level (biotinylation assay) as WT at 28 and 37 °C.

**W887R**	missense mutation	FHM	TM7-8 loop	(De Fusco et al., 2003)	(De Fusco et al., 2003)<br>(Capendeguy and Horisberger, 2004)<br>(Koenderink et al., 2005)<br>(Leo et al., 2011)	COS7 cells: no cell survival under ouabain challenge, normal *in vitro* protein synthesis, physiological location of and mutant protein in the membrane fraction<br>*X. laevis* oocytes: strongly reduced expression in pulse chase labeling experiment, no ouabain binding; homologous *Bufo marinus* α1 mutant: no palytoxin-induced current, no pump current, suspicious K^+-induced outward current in the absence of external Na^+<br>*X. laevis* oocytes: Plasma membrane expression similar to WT, ouabain binding strongly reduced, ATPase activity strongly reduced (oocyte total membranes), Rb^+ uptake strongly reduced; electrophysiology: strongly reduced pump currents<br>Transgenic mice: homozygous ATP1A2(W887R/W887R) mutants die shortly after birth, heterozygous ATP1A2(+/W887R) mutants show no apparent phenotype but enhanced susceptibility for CSD; no or strongly reduced ATP1A2 protein in homo- or heterozygous mutants brains, respectively<br>HeLa cells: decreased W887R mutant protein; immunofluorescence: ER localisation dominant (no plasma membrane protein), pattern changed by proteasome inhibitors

**G900R**	missense mutation	FHM & epilepsy	TM7-8 loop	(Deprez et al., 2008)	(Spiller and Friedrich, 2014) (Swarts et al., 2013)	*X. laevis* oocytes: normal K^+ and voltage dependence of pump currents, voltage dependence of ouabain-sensitive Na^+/Na^+ exchange currents similar to WT, general increase in rate constants of Na^+/Na^+ exchange currents<br>Sf9 cells: normal protein expression, strongly reduced ouabain binding with increased ouabain affinity; apparent K^+ and Na^+ affinity as WT, reduced ATPase activity with decreased ATP affinity and normal turnover in ATPase assay compared to WT
**E902K**	missense mutation	FHM	TM7-8, loop	(Jurkat-Rott et al., 2004)	(Spiller and Friedrich, 2014) (Swarts et al., 2013)	*X. laevis* oocytes: normal pump currents with slightly increased apparent K^+ affinity at positive potentials, voltage dependence of ouabain-sensitive Na^+/Na^+ exchange currents similar to WT, general increase in rate constants of Na^+/Na^+ exchange currents<br>Sf9 cells: normal protein expression, strongly reduced ouabain binding and slightly increased ouabain affinity; slightly decreased apparent Na^+ similar apparent K+ affinity, reduced ATPase activity with decreased ATP affinity and normal turnover in ATPase assay compared to WT
**R908Q**	missense mutation	SHM, FHM	TM7-8 loop	(de Vries et al., 2007) (Hermann et al., 2013) (Roth et al., 2014)	(de Vries et al., 2007) (Tavraz et al., 2009)	HeLa cells: no survival under ouabain challenge<br>*X. laevis* oocytes: reduced Rb^+ uptake, reduced pump currents; K^+ and voltage dependence of pump currents, kinetics and voltage dependence of ouabain-sensitive Na^+/Na^+ exchange currents similar to WT; normal overall and reduced plasma membrane protein level
**H916L**	missense mutation	FHM with prolonged aura	TM8	(Iizuka et al., 2014)		
**Q927P**	missense mutation	FHM + epilepsy (generalized seizures)	TM8	(Riant et al., 2010)		

**del(K935-S940)insI**	deletion & insertion	FHM	TM8-9 loop	(Riant et al., 2005)	(Tavraz et al., 2008) (Weigand et al., 2014)	*X. laevis* oocytes: no pump currents, no Rb^+ uptake, normal overall but strongly reduced plasma membrane protein level<br>Sf9 cells: normal protein expression, no ouabain binding, no ATPase activity
**R937P**	missense mutation	FHM	TM8-9 loop	(Riant et al., 2005)	(Tavraz et al., 2008) (Poulsen et al., 2010) (Weigand et al., 2014)	*X. laevis* oocytes: no pump currents, strongly reduced Rb^+ uptake, normal overall and plasma membrane protein level<br>*X. laevis* oocytes: extreme negative shift of voltage dependence of ouabain-sensitive Na^+/Na^+ exchange currents (↔decreased apparent affinity for extracellular Na^+), inverted voltage dependence of rate constants of Na^+/Na^+ exchange currents (high at positive, low at negative voltages), strongly augmented “leak” currents in the presence of extracellular Na^+<br>Sf9 cells: normal protein expression, strongly reduced ouabain binding, no ATPase activity
**S940L**	Missense mutation	FHM & pulmonary arterial hypertension	TM9	(Montani et al., 2013)		
**S966fs**	frameshift, premature Stop	FHM	TM9-10 loop	(Riant et al., 2005)	(Tavraz et al., 2008) this study	*X. laevis* oocytes: no pump currents, no Rb^+ uptake, normal overall protein expression but hardly any plasma membrane protein

**P979L**	missense mutation	FHM, SHM & reversible cerebral vasoconstriction	TM9-10 loop	(Jurkat-Rott et al., 2004)<br>(Hermann et al., 2013)	(Tavraz et al., 2008)<br>(Tavraz et al., 2008)<br>this work	*X. laevis* oocytes: K^+ and voltage dependence of pump currents, kinetics and voltage dependence of ouabain-sensitive Na^+/Na^+ exchange currents as WT; normal overall and plasma membrane protein level<br>HEK293FT cells: overall and plasma membrane protein level (biotinylation assay) as WT at 28 °C, strongly reduced plasma membrane protein level at 37 °C<br>HEK293T cells, fluorescence microscopy: Redistribution of mutant plasma membrane protein from normal plasma membrane expression at 28 °C to strongly reduced plasma membrane protein at 37 °C (this work)
**L994del**	deletion	SHM + epilepsy (focal seizures)	TM10	(Riant et al., 2010)	(Spiller and Friedrich, 2014)	*X. laevis* oocytes: no pump currents, normal overall protein expression but strongly reduced plasma membrane protein
**D999H**	missense mutation	FHM, acute enzephalopathy	C-Terminus	(Fernandez et al., 2008)<br>(Merwick et al., 2013)	(Spiller and Friedrich, 2014)<br>(Weigand et al., 2014)	*X. laevis* oocytes: strongly augmented voltage-dependent decrease of pump currents at voltages below +20 mV and strongly altered K^+ dependence of pump currents with increased apparent affinity for K^+ below 20 mV and decreased apparent K^+ affinity above 0 mV; strongly reduced voltage dependence with high an essentially voltage-insensitive rate constants of ouabain-sensitive Na^+/Na^+ exchange currents<br>Sf9 cells: normal protein expression, no ouabain binding, no ATPase activity
**R1002Q**	missense mutation	FHM	C-Terminus	(Jen et al., 2007)	(Jen et al., 2007)<br>(Poulsen et al., 2010)	HeLa cells: strongly reduced survival under ouabain challenge<br>*X. laevis* oocytes: strong negative shift of voltage dependence of ouabain-sensitive Na^+/Na^+ exchange currents (↔decreased apparent affinity for extracellular Na^+), inverted voltage dependence of rate constants of Na^+/Na^+ exchange currents (high at positive, low at negative voltages)

**K1003E**	missense mutation	SHM + epilepsy (focal seizures)	C-Terminus	(Riant et al., 2010)	(Spiller and Friedrich, 2014)	*X. laevis* oocytes: normal K^+ and voltage dependence of pump currents, negative shift of voltage dependence of ouabain-sensitive Na^+/Na^+ exchange currents (↔decreased apparent affinity for extracellular Na^+), increase in rate constants of Na^+/Na^+ exchange currents above -40 mV
**R1007W**	missense mutation	FHM	C-Terminus	(Pisano et al., 2013)	(Pisano et al., 2013)	*X. laevis* oocytes: electrophysiology: pump current amplitudes identical to WT, apparent K^+ affinity above -60 mV increased compared to WT, positive shift of voltage dependence (↔increased apparent affinity for extracellular Na^+)and changed voltage-dependent kinetics of ouabain-sensitive Na^+/Na^+ exchange currents (increase of rate constants) compared to WT
**Y1009X**	non-sense mutation	SHM	C-Terminus	(Gallanti et al., 2011) (Gallanti et al., 2008)	(Spiller and Friedrich, 2014)	*X. laevis* oocytes: no pump currents, normal overall protein expression but strongly reduced plasma membrane protein

**X1021R**	read-through mutation, C-terminal extension by 28 AA	FHM	C-Terminus Stop Codon	(Jurkat-Rott et al., 2004)	(Tavraz et al., 2008) (Toustrup-Jensen et al., 2014)	*X. laevis* oocytes: reduced pump currents, strongly reduced turnover, normal overall and plasma membrane protein level, strongly increased apparent affinity for K^+ from pump currents, drastically changed voltage dependence of ouabain-sensitive Na^+/Na^+ exchange currents compared to WT (strong negative shift↔decreased apparent affinity for extracellular Na^+; strongly reduced equivalent charge), voltage dependence of rate constants from Na^+/Na^+ exchange currents opposite compared to WT COS cells: 28 amino acid extension in human ATP1A2 was inable to achieve stable cell lines under ouabain selection conditions, no ATP-dependent phosphorylation in transient transfection experiments; 28 amino acid extension in rat ATP1A1 permitted generation of stable cell line under ouabain selection conditions, strongly reduced apparent Na^+ affinity, similar apparent K^+ affinity (but higher at high Na^+), increased ATP and decreased vanadate sensitivity, reduced ouabain affinity compared to WT

References

Aceves, J., Mungall, D., and Kirmani, B.F. (2013). Sporadic Hemiplegic Migraine with ATP1A2 and Prothrombin Gene Mutations. *Case Rep. Neurol. Med.* 2013**,** 895057.

Al-Bulushi, B., Al-Hashem, A., and Tabarki, B. (2014). A wide clinical phenotype spectrum in patients with ATP1A2 mutations. *J. Child Neurol.* 29**,** 265-268.

Ambrosini, A., D'Onofrio, M., Grieco, G.S., Di Mambro, A., Montagna, G., Fortini, D., Nicoletti, F., Nappi, G., Sances, G., Schoenen, J., Buzzi, M.G., Santorelli, F.M., and Pierelli, F. (2005). Familial basilar migraine associated with a new mutation in the ATP1A2 gene. *Neurology* 65**,** 1826-1828.

Asghar, S.J., Milesi-Halle, A., Kaushik, C., Glasier, C., and Sharp, G.B. (2012). Variable manifestations of familial hemiplegic migraine associated with reversible cerebral edema in children. *Pediatr. Neurol.* 47**,** 201-204.

Barros, J., Mendes, A., Matos, I., and Pereira-Monteiro, J. (2012). Psychotic aura symptoms in familial hemiplegic migraine type 2 (ATP1A2). *J. Headache Pain* 13**,** 581-585.

Bassi, M.T., Bresolin, N., Tonelli, A., Nazos, K., Crippa, F., Baschirotto, C., Zucca, C., Bersano, A., Dolcetta, D., Boneschi, F.M., Barone, V., and Casari, G. (2004). A novel mutation in the ATP1A2 gene causes alternating hemiplegia of childhood. *J. Med. Genet.* 41**,** 621-628.

Capendeguy, O., and Horisberger, J.D. (2004). Functional effects of Na^{+},K^{+}-ATPase gene mutations linked to familial hemiplegic migraine. *Neuromolecular Med.* 6**,** 105-116.

Carreño, O., Corominas, R., Serra, S.A., Sintas, C., Fernandez-Castillo, N., Vila-Pueyo, M., Toma, C., Gene, G.G., Pons, R., Llaneza, M., Sobrido, M.J., Grinberg, D., Valverde, M.A., Fernandez-Fernandez, J.M., Macaya, A., and Cormand, B. (2013). Screening of CACNA1A and ATP1A2 genes in hemiplegic migraine: clinical, genetic, and functional studies. *Mol. Genet. Genomic Med.* 1**,** 206-222.

Castro, M.J., Lemos, C., Barros, J., Vanmolkot, K.R., van den Heuvel, J.J., Koenderink, J.B., Maciel, P., Pereira-Monteiro, J.M., van den Maagdenberg, A.M., and Sequeiros, J. (2008a). "Mutation screening for CACNA1A and ATP1A2 FHM genes in a set of migraine probands: identification of a novel rare R51H variant in ATP1A2," in *Genetics in the Identification of Molecular Pathogenic Mechanisms of Familial Migraine (PhD Thesis, Instituto de Ciências Biomédicas de Abel Salazar da Universidade do Porto),* ed. M.J. Castro. (Porto), 107-116.

Castro, M.J., Nunes, B., de Vries, B., Lemos, C., Vanmolkot, K.R., van den Heuvel, J.J., Temudo, T., Barros, J., Sequeiros, J., Frants, R.R., Koenderink, J.B., Pereira-Monteiro, J.M., and van den Maagdenberg, A.M. (2008b). Two novel functional mutations in the Na^{+},K^{+}-ATPase alpha2-subunit ATP1A2 gene in patients with familial hemiplegic migraine and associated neurological phenotypes. *Clin. Genet.* 73**,** 37-43.

Castro, M.J., Stam, A.H., Lemos, C., Barros, J., Gouveia, R.G., Martins, I.P., Koenderink, J.B., Vanmolkot, K.R., Mendes, A.P., Frants, R.R., Ferrari, M.D., Sequeiros, J., Pereira-Monteiro, J.M., and van den Maagdenberg, A.M. (2007). Recurrent ATP1A2 mutations in Portuguese families with familial hemiplegic migraine. *J. Hum. Genet.* 52**,** 990-998.

Costa, C., Prontera, P., Sarchielli, P., Tonelli, A., Bassi, M.T., Cupini, L.M., Caproni, S., Siliquini, S., Donti, E., and Calabresi, P. (2014). A novel ATP1A2 gene mutation in familial hemiplegic migraine and epilepsy. *Cephalalgia* 34**,** 68-72.

De Cunto, A., Bensa, M., and Tonelli, A. (2012). A case of familial hemiplegic migraine associated with a novel ATP1A2 gene mutation. *Pediatr. Neurol.* 47**,** 133-136.

De Fusco, M., Marconi, R., Silvestri, L., Atorino, L., Rampoldi, L., Morgante, L., Ballabio, A., Aridon, P., and Casari, G. (2003). Haploinsufficiency of ATP1A2 encoding the

Na^+/K^+ pump alpha2 subunit associated with familial hemiplegic migraine type 2. *Nat. Genet.* 33**,** 192-196.

De Sanctis, S., Grieco, G.S., Breda, L., Casali, C., Nozzi, M., Del Torto, M., Chiarelli, F., and Verrotti, A. (2011). Prolonged sporadic hemiplegic migraine associated with a novel de novo missense ATP1A2 gene mutation. *Headache* 51**,** 447-450.

de Vries, B., Freilinger, T., Vanmolkot, K.R., Koenderink, J.B., Stam, A.H., Terwindt, G.M., Babini, E., van den Boogerd, E.H., van den Heuvel, J.J., Frants, R.R., Haan, J., Pusch, M., van den Maagdenberg, A.M., Ferrari, M.D., and Dichgans, M. (2007). Systematic analysis of three FHM genes in 39 sporadic patients with hemiplegic migraine. *Neurology* 69**,** 2170-2176.

de Vries, B., Stam, A.H., Kirkpatrick, M., Vanmolkot, K.R., Koenderink, J.B., van den Heuvel, J.J., Stunnenberg, B., Goudie, D., Shetty, J., Jain, V., van Vark, J., Terwindt, G.M., Frants, R.R., Haan, J., van den Maagdenberg, A.M., and Ferrari, M.D. (2009). Familial hemiplegic migraine is associated with febrile seizures in an FHM2 family with a novel de novo ATP1A2 mutation. *Epilepsia* 50**,** 2503-2504.

Deprez, L., Weckhuysen, S., Peeters, K., Deconinck, T., Claeys, K.G., Claes, L.R., Suls, A., Van Dyck, T., Palmini, A., Matthijs, G., Van Paesschen, W., and De Jonghe, P. (2008). Epilepsy as part of the phenotype associated with ATP1A2 mutations. *Epilepsia* 49**,** 500-508.

Fernandez, D.M., Hand, C.K., Sweeney, B.J., and Parfrey, N.A. (2008). A novel ATP1A2 gene mutation in an Irish familial hemiplegic migraine kindred. *Headache* 48**,** 101-108.

Gallanti, A., Cardin, V., Tonelli, A., Bussone, G., Bresolin, N., Mariani, C., and Bassi, M.T. (2011). The genetic features of 24 patients affected by familial and sporadic hemiplegic migraine. *Neurol. Sci.* 32 Suppl 1**,** S141-142.

Gallanti, A., Tonelli, A., Cardin, V., Bussone, G., Bresolin, N., and Bassi, M.T. (2008). A novel de novo nonsense mutation in ATP1A2 associated with sporadic hemiplegic migraine and epileptic seizures. *J. Neurol. Sci.* 273**,** 123-126.

Hermann, A., Engelandt, K., Rautenstrauss, B., Reichmann, H., and Jacobasch, E. (2013). Hemiplegic migraine with reversible cerebral vasoconstriction caused by ATP1A2 mutations. *J. Neurol.* 260**,** 2172-2174.

Iizuka, T., Takahashi, Y., Sato, M., Yonekura, J., Miyakawa, S., Endo, M., Hamada, J., Kan, S., Mochizuki, H., Momose, Y., Tsuji, S., and Sakai, F. (2014). Neurovascular changes in prolonged migraine aura in FHM with a novel ATP1A2 gene mutation. *J. Neurol Neurosurg. Psychiatry* 83**,** 205-212.

Jen, J.C., Klein, A., Boltshauser, E., Cartwright, M.S., Roach, E.S., Mamsa, H., and Baloh, R.W. (2007). Prolonged hemiplegic episodes in children due to mutations in ATP1A2. *J. Neurol. Neurosurg. Psychiatry* 78**,** 523-526.

Jurkat-Rott, K., Freilinger, T., Dreier, J.P., Herzog, J., Göbel, H., Petzold, G.C., Montagna, P., Gasser, T., Lehmann-Horn, F., and Dichgans, M. (2004). Variability of familial hemiplegic migraine with novel A1A2 Na^+/K^+-ATPase variants. *Neurology* 62**,** 1857-1861.

Kaunisto, M.A., Harno, H., Vanmolkot, K.R., Gargus, J.J., Sun, G., Hamalainen, E., Liukkonen, E., Kallela, M., van den Maagdenberg, A.M., Frants, R.R., Farkkila, M., Palotie, A., and Wessman, M. (2004). A novel missense ATP1A2 mutation in a Finnish family with familial hemiplegic migraine type 2. *Neurogenetics* 5**,** 141-146.

Koenderink, J.B., Zifarelli, G., Qiu, L.Y., Schwarz, W., De Pont, J.J., Bamberg, E., and Friedrich, T. (2005). Na,K-ATPase mutations in familial hemiplegic migraine lead to functional inactivation. *Biochim. Biophys. Acta* 1669**,** 61-68.

Lebas, A., Guyant-Marechal, L., Hannequin, D., Riant, F., Tournier-Lasserve, E., and Parain, D. (2008). Severe attacks of familial hemiplegic migraine, childhood epilepsy and ATP1A2 mutation. *Cephalalgia* 28**,** 774-777.

Leo, L., Gherardini, L., Barone, V., De Fusco, M., Pietrobon, D., Pizzorusso, T., and Casari, G. (2011). Increased susceptibility to cortical spreading depression in the mouse model of familial hemiplegic migraine type 2. *PLoS Genet.* 7**,** e1002129.

Merwick, A., Fernandez, D., McNamara, B., and Harrington, H. (2013). Acute encephalopathy in familial hemiplegic migraine with ATP1A2 mutation. *BMJ Case Rep.* 2013.

Montani, D., Girerd, B., Gunther, S., Riant, F., Tournier-Lasserve, E., Magy, L., Maazi, N., Guignabert, C., Savale, L., Sitbon, O., Simonneau, G., Soubrier, F., and Humbert, M. (2013). Pulmonary arterial hypertension in familial hemiplegic migraine with ATP1A2 channelopathy. *Eur. Respir. J.* 43**,** 641-643.

Oh, S.K., Baek, J.I., Weigand, K.M., Venselaar, H., Swarts, H.G., Park, S.H., Hashim Raza, M., Jung da, J., Choi, S.Y., Lee, S.H., Friedrich, T., Vriend, G., Koenderink, J.B., Kim, U.K., and Lee, K.Y. (2015). A missense variant of the ATP1A2 gene is associated with a novel phenotype of progressive sensorineural hearing loss associated with migraine. *Eur. J. Hum. Genet.* 23**,** 639-645.

Pierelli, F., Grieco, G.S., Pauri, F., Pirro, C., Fiermonte, G., Ambrosini, A., Costa, A., Buzzi, M.G., Valoppi, M., Caltagirone, C., Nappi, G., and Santorelli, F.M. (2006). A novel ATP1A2 mutation in a family with FHM type II. *Cephalalgia* 26**,** 324-328.

Pisano, T., Spiller, S., Mei, D., Guerrini, R., Cianchetti, C., Friedrich, T., and Pruna, D. (2013). Functional characterization of a novel C-terminal ATP1A2 mutation causing hemiplegic migraine and epilepsy. *Cephalalgia* 33**,** 1302-1310.

Podestà, B., Briatore, E., Boghi, A., Marenco, D., and Calzolari, S. (2011). Transient nonverbal learning disorder in a child suffering from Familial Hemiplegic Migraine. *Cephalalgia* 31**,** 1497-1502.

Poulsen, H., Khandelia, H., Morth, J.P., Bublitz, M., Mouritsen, O.G., Egebjerg, J., and Nissen, P. (2010). Neurological disease mutations compromise a C-terminal ion pathway in the Na^+/K^+-ATPase. *Nature* 467**,** 99-102.

Price, E.M., and Lingrel, J.B. (1988). Structure-function relationships in the Na,K-ATPase alpha subunit: site-directed mutagenesis of glutamine-111 to arginine and asparagine-122 to aspartic acid generates a ouabain-resistant enzyme. *Biochemistry* 27**,** 8400-8408.

Riant, F., De Fusco, M., Aridon, P., Ducros, A., Ploton, C., Marchelli, F., Maciazek, J., Bousser, M.G., Casari, G., and Tournier-Lasserve, E. (2005). ATP1A2 mutations in 11 families with familial hemiplegic migraine. *Hum. Mutat.* 26**,** 281.

Riant, F., Ducros, A., Ploton, C., Barbance, C., Depienne, C., and Tournier-Lasserve, E. (2010). De novo mutations in ATP1A2 and CACNA1A are frequent in early-onset sporadic hemiplegic migraine. *Neurology* 75**,** 967-972.

Roth, C., Freilinger, T., Kirovski, G., Dunkel, J., Shah, Y., Wilken, B., Rautenstrauss, B., and Ferbert, A. (2014). Clinical spectrum in three families with familial hemiplegic migraine type 2 including a novel mutation in the ATP1A2 gene. *Cephalalgia* 34**,** 183-190.

Santoro, L., Manganelli, F., Fortunato, M.R., Soldovieri, M.V., Ambrosino, P., Iodice, R., Pisciotta, C., Tessa, A., Santorelli, F., and Taglialatela, M. (2011). A new Italian FHM2 family: clinical aspects and functional analysis of the disease-associated mutation. *Cephalalgia* 31**,** 808-819.

Schack, V.R., Holm, R., and Vilsen, B. (2012). Inhibition of phosphorylation of Na^+,K^+-ATPase by mutations causing familial hemiplegic migraine. *J. Biol. Chem.* 287**,** 2191-2202.

Schmitt, F.J., Thaa, B., Junghans, C., Vitali, M., Veit, M., and Friedrich, T. (2014). eGFP-pHsens as a highly sensitive fluorophore for cellular pH determination by fluorescence lifetime imaging microscopy (FLIM). *Biochim. Biophys. Acta* 1837**,** 1581-1593.

Segall, L., Mezzetti, A., Scanzano, R., Gargus, J.J., Purisima, E., and Blostein, R. (2005). Alterations in the alpha2 isoform of Na,K-ATPase associated with familial hemiplegic migraine type 2. *Proc. Natl. Acad. Sci. U. S. A.* 102**,** 11106-11111.

Segall, L., Scanzano, R., Kaunisto, M.A., Wessman, M., Palotie, A., Gargus, J.J., and Blostein, R. (2004). Kinetic alterations due to a missense mutation in the Na,K-ATPase alpha2 subunit cause familial hemiplegic migraine type 2. *J. Biol. Chem.* 279**,** 43692-42696.

Spadaro, M., Ursu, S., Lehmann-Horn, F., Veneziano, L., Antonini, G., Giunti, P., Frontali, M., and Jurkat-Rott, K. (2004). A G301R Na^+/K^+ -ATPase mutation causes familial hemiplegic migraine type 2 with cerebellar signs. *Neurogenetics* 5**,** 177-185.

Spiller, S., and Friedrich, T. (2014). Functional analysis of human Na^+/K^+-ATPase familial or sporadic hemiplegic migraine mutations expressed in *Xenopus* oocytes. *World J. Biol. Chem.* 5**,** 240-253.

Swarts, H.G., Weigand, K.M., Venselaar, H., van den Maagdenberg, A.M., Russel, F.G., and Koenderink, J.B. (2013). Familial hemiplegic migraine mutations affect Na,K-ATPase domain interactions. *Biochim. Biophys. Acta* 1832**,** 2173-2179.

Swoboda, K.J., Kanavakis, E., Xaidara, A., Johnson, J.E., Leppert, M.F., Schlesinger-Massart, M.B., Ptacek, L.J., Silver, K., and Youroukos, S. (2004). Alternating hemiplegia of childhood or familial hemiplegic migraine? A novel ATP1A2 mutation. *Ann. Neurol.* 55**,** 884-887.

Tavraz, N.N., Dürr, K.L., Koenderink, J.B., Freilinger, T., Bamberg, E., Dichgans, M., and Friedrich, T. (2009). Impaired plasma membrane targeting or protein stability by certain ATP1A2 mutations identified in sporadic or familial hemiplegic migraine. *Channels (Austin)* 3**,** 82-87.

Tavraz, N.N., Friedrich, T., Dürr, K.L., Koenderink, J.B., Bamberg, E., Freilinger, T., and Dichgans, M. (2008). Diverse functional consequences of mutations in the Na^+/K^+-ATPase alpha2-subunit causing familial hemiplegic migraine type 2. *J. Biol. Chem.* 283**,** 31097-31106.

Thomsen, L.L., Kirchmann, M., Bjornsson, A., Stefansson, H., Jensen, R.M., Fasquel, A.C., Petursson, H., Stefansson, M., Frigge, M.L., Kong, A., Gulcher, J., Stefansson, K., and Olesen, J. (2007). The genetic spectrum of a population-based sample of familial hemiplegic migraine. *Brain* 130**,** 346-356.

Thomsen, L.L., Oestergaard, E., Bjornsson, A., Stefansson, H., Fasquel, A.C., Gulcher, J., Stefansson, K., and Olesen, J. (2008). Screen for CACNA1A and ATP1A2 mutations in sporadic hemiplegic migraine patients. *Cephalalgia* 28**,** 914-921.

Todt, U., Dichgans, M., Jurkat-Rott, K., Heinze, A., Zifarelli, G., Koenderink, J.B., Goebel, I., Zumbroich, V., Stiller, A., Ramirez, A., Friedrich, T., Gobel, H., and Kubisch, C. (2005). Rare missense variants in ATP1A2 in families with clustering of common forms of migraine. *Hum. Mutat.* 26**,** 315-321.

Toldo, I., Cecchin, D., Sartori, S., Calderone, M., Mardari, R., Cattelan, F., Laverda, A.M., Drigo, P., and Battistella, P.A. (2010). Multimodal neuroimaging in a child with sporadic hemiplegic migraine: a contribution to understanding pathogenesis. *Cephalalgia* 31**,** 751-756.

Tonelli, A., Gallanti, A., Bersano, A., Cardin, V., Ballabio, E., Airoldi, G., Redaelli, F., Candelise, L., Bresolin, N., and Bassi, M.T. (2007). Amino acid changes in the amino terminus of the Na,K-adenosine triphosphatase alpha-2 subunit associated to familial and sporadic hemiplegic migraine. *Clin. Genet.* 72**,** 517-523.

Toustrup-Jensen, M.S., Einholm, A.P., Schack, V.R., Nielsen, H.N., Holm, R., Sobrido, M.J., Andersen, J.P., Clausen, T., and Vilsen, B. (2014). Relationship between intracellular Na^+ concentration and reduced Na^+ affinity in Na^+,K^+-ATPase mutants causing neurological disease. *J. Biol. Chem.* 289**,** 3186-3197.

Vanmolkot, K.R., Kors, E.E., Hottenga, J.J., Terwindt, G.M., Haan, J., Hoefnagels, W.A., Black, D.F., Sandkuijl, L.A., Frants, R.R., Ferrari, M.D., and van den Maagdenberg, A.M. (2003). Novel mutations in the Na^+, K^+-ATPase pump gene ATP1A2 associated with familial hemiplegic migraine and benign familial infantile convulsions. *Ann. Neurol.* 54**,** 360-366.

Vanmolkot, K.R., Kors, E.E., Turk, U., Turkdogan, D., Keyser, A., Broos, L.A., Kia, S.K., van den Heuvel, J.J., Black, D.F., Haan, J., Frants, R.R., Barone, V., Ferrari, M.D., Casari, G., Koenderink, J.B., and van den Maagdenberg, A.M. (2006a). Two de novo mutations in the Na,K-ATPase gene ATP1A2 associated with pure familial hemiplegic migraine. *Eur. J. Hum. Genet.* 14**,** 555-560.

Vanmolkot, K.R., Stam, A.H., Raman, A., Koenderink, J.B., de Vries, B., van den Boogerd, E.H., van Vark, J., van den Heuvel, J.J., Bajaj, N., Terwindt, G.M., Haan, J., Frants, R.R., Ferrari, M.D., and van den Maagdenberg, A.M. (2007). First case of compound heterozygosity in Na,K-ATPase gene ATP1A2 in familial hemiplegic migraine. *Eur. J. Hum. Genet.* 15**,** 884-888.

Vanmolkot, K.R., Stroink, H., Koenderink, J.B., Kors, E.E., van den Heuvel, J.J., van den Boogerd, E.H., Stam, A.H., Haan, J., De Vries, B.B., Terwindt, G.M., Frants, R.R., Ferrari, M.D., and van den Maagdenberg, A.M. (2006b). Severe episodic neurological deficits and permanent mental retardation in a child with a novel FHM2 ATP1A2 mutation. *Ann. Neurol.* 59**,** 310-314.

Weigand, K.M., Swarts, H.G., Russel, F.G., and Koenderink, J.B. (2014). Biochemical characterization of sporadic/familial hemiplegic migraine mutations. *Biochim. Biophys. Acta* 1838**,** 1693-1700.
